# Quantum Hall antidot as a fractional coulombmeter

**DOI:** 10.1038/s41567-026-03412-2

**Published:** 2026-08-14

**Authors:** Mario Di Luca, Emily Hajigeorgiou, Zekang Zhou, Tevž Lotrič, Tengyan Feng, Kenji Watanabe, Takashi Taniguchi, Steven H. Simon, Mitali Banerjee

**Affiliations:** 1 Institute of Physics, École Polytechnique Fédérale de Lausanne (EPFL), Lausanne, Switzerland; 2 Rudolf Peierls Centre for Theoretical Physics, Oxford, UK; 3 Research Center for Functional Materials, National Institute for Materials Science, Tsukuba, Japan; 4 International Center for Materials Nanoarchitectonics, National Institute for Materials Science, Tsukuba, Japan; 5 Center for Quantum Science and Engineering (QSE Center), École Polytechnique Fédérale de Lausanne (EPFL), Lausanne, Switzerland

**Keywords:** Quantum Hall, Electronic properties and materials

## Abstract

The ability to detect the fractionally charged quasiparticles that arise in the fractional quantum Hall regime is of fundamental importance for probing their quantum properties. Although electronic interferometers have successfully probed their statistical properties, interpreting the results is often complicated by bulk–edge interactions. Antidots—tunable hills in the potential landscape in the device—are particularly valuable in this context, as they overcome the geometric limitations of conventional interferometer designs and act as controlled impurities. Here we demonstrate a method for extracting the charge of quasiparticles as they tunnel through an antidot. To accomplish this, we employ a gate-defined bilayer-graphene antidot operating in the Coulomb-dominated regime to study quasiparticle tunnelling in both integer and fractional quantum Hall states and we report direct measurements of fractional charge. Our derived theoretical model indicates that the differences in the measured charges may be attributed to variations in edge re-equilibration arising from a different parity of downstream integer edge modes. The simplicity and tunability of this design open a pathway to extending antidot-based charge measurements to other van der Waals materials and establishing antidots as a broadly applicable platform for studying topological materials.

## Abstract (web summary)

A transport technique that allows the measurement of the effective charge of fractional quantum Hall states as they tunnel through a controlled impurity is demonstrated.

## Main

The fractional quantum Hall (FQH) effect provides a versatile platform for exploring topological states of matter. It is characterized by the presence of fractionally charged quasiparticles. These excitations, known as anyons, carry fractional charge and exhibit quantum statistics distinct from those of bosons and fermions^1^. Braiding experiments using electronic interferometers^2^ provide a direct probe of anyonic statistics. The braiding of abelian anyons appears as phase jumps in Aharonov–Bohm oscillations^3,4^. Such phase jumps were first observed at filling factor *ν* = 1/3 in GaAs Fabry–Pérot interferometers^5,6^ and more recently in graphene-based Fabry–Pérot interferometers^7–9^. However, in these experiments, the phase jumps occurred stochastically, as they were caused by quasiparticle fluctuations in the bulk, and it was not possible to control them. Recent experiments applying a chiral Mach–Zehnder interferometer in GaAs, which incorporates an antidot (AD) to control the number of bulk quasiparticles, have demonstrated that phase jumps occur concurrently with a change in the number of bulk quasiparticles^10,11^. Although these results are important for particle-like FQH states (for example, *ν* = 1/3), the presence of bulk–edge interactions still obscures the outcomes in hole-like FQH states (for example, *ν* = 2/3)^11,12^. These complications, therefore, limit the use of Fabry–Pérot interferometers and chiral Mach–Zehnder interferometers for probing non-abelian quasiparticles in exotic states such as *ν* = 5/2 (refs. ^13–15^).

Another possible geometry is the quantum Hall AD, in which anyons are localized around a potential hill^16–18^. ADs have been extensively studied in GaAs heterostructures in the integer quantum Hall (IQH) regime^19–23^, and they played an important role in the first detection of fractionally charged quasiparticles^24–27^. Although interference measurements are essential, a complete understanding of anyonic physics also requires the ability to manipulate individual quasiparticles and directly verify their fractional charge e^*^. In this context, ADs are predicted to be an ideal platform for studying non-abelian states^28,29^ and controllably braiding non-abelian quasiparticles^30,31^.

Despite its simple geometry, not much has been explored in graphene due to the challenges of fabricating high-quality tunable ADs in van der Waals heterostructures. The few efforts with monolayer graphene have been limited to the study of localized edges via scanning tunnelling microscopy^32–34^ or scanning gate microscopy^35^ and lithographically etched structures in hexagonal boron nitride (hBN)-encapsulated or suspended graphene^36,37^. However, these approaches lack the tunability needed to control the coupling between the extended edges and the AD-bound states.

Here we present a dual-gate-defined bilayer-graphene AD operating in the Coulomb-dominated regime as a platform for measuring the charge of a quasiparticle tunnelling through the fractional bulk. We observed Coulomb-dominated oscillations at both integer and fractional filling factors, and we extracted the quasiparticle charge directly from the conductance oscillations as a function of carrier density and magnetic field. In particular, at filling factors 4/3 and 7/3, we identified tunnelling quasiparticles with charge *e*/3. However, at the hole-conjugate filling factors, such as 2/3, 5/3 and 8/3, we measured different quasiparticle charges. Together with theoretical calculations, our results reveal that, although these hole-conjugate states appear nominally similar, they differ because of the presence of the number of integer edge modes, which lead to distinct tunnelling charges. Our findings are consistent with previous measurements in GaAs heterostructures for the lowest Landau level (LL)^27^, while revealing distinct behaviours for the higher LLs that have not been previously studied.

## Device description

An AD is a potential hill introduced into a two-dimensional electron gas in a perpendicular magnetic field. In the quantum Hall regime, chiral edge modes encircle the AD while extended channels carry current along the device boundaries. The main effect of the AD potential is to lift the degeneracy of the LLs encircling it by splitting the energy spectrum into quantized levels, each hosting one charge carrier^17,33,34^. The energy splitting is related to the velocity of the edge mode *v* and the diameter of the AD *D*_AD_, δ*ϵ* ∝ *ℏv*/*D*_AD_ (ref. ^36^), where *h* is the Planck constant. By tuning an external parameter, such as the magnetic field, the top-gate (TG) or bottom-gate (BG) voltages, it is possible to control the number of quasiparticles confined in the AD-bound states and to detect them through resonant tunnelling between the extended edge channels and the quantized AD-bound states.

**Fig. 1:**
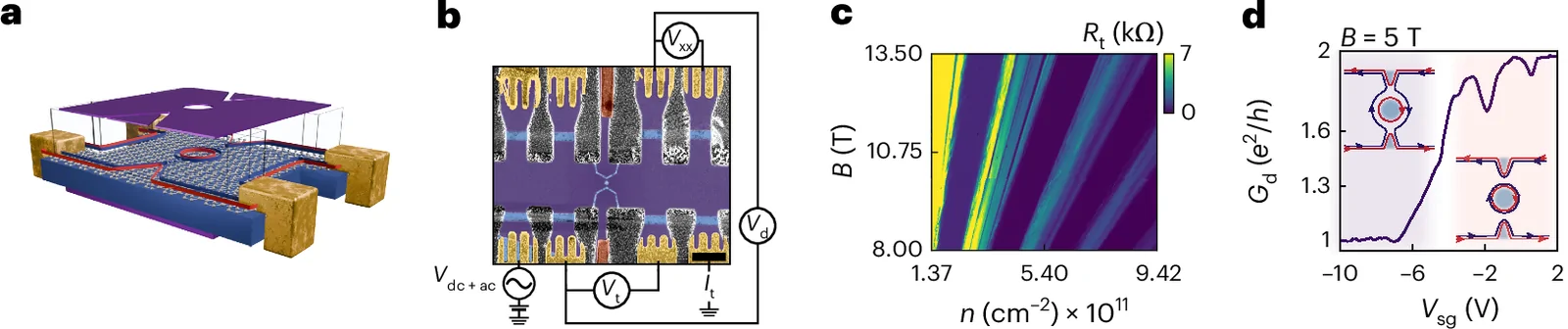
Gate-defined AD in bilayer-graphene. **a**, Schematic of the device at *ν* = 2 showing the interaction between the inner extended edge and AD-bound states (blue). The outer edge state (red) is fully transmitted. The graphite gates are shown in purple, and the bottom hBN in blue. Only the edges of the top hBN are shown for clarity. **b**, False-colour scanning electron microscope image of an AD device. The ohmic contacts are shown in gold, the palladium contacts to the graphite TG in dark orange and the graphite in purple. The top hBN (blue) is visible in places where the top graphite has been etched. The AD area is defined by the etched circle in the top graphite. A voltage *V*_ac_ = 10 μV (3 μV) was applied for the integer (fractional) oscillations, and the transmitted *I*_t_ current was measured. The voltage was measured across the AD *V*_t_ and diagonally *V*_d_ as well as on the side of it *V*_xx_. **c**, Fan diagram of the transmitted resistance *R*_t_ measured across the AD as a function of the carrier density *n* and the magnetic field *B*. **d**, Diagonal conductance *G*_d_ for *ν* = 2 as a function of the SG voltage *V*_sg_. As *V*_sg_ decreases, the inner edge can be selectively partitioned, and it crosses over from full transmission (*V*_sg_ ≈ 2 V) to full reflection (*V*_sg_ ≈ −8 V). Each inset schematically represents the system at the corresponding shaded voltage range. Scale bar: 2 μm.

In Fig. 1a, we present our gate-defined bilayer-graphene AD device. The AD is electrostatically defined by an etched hole in the top graphite gate (in our devices, the electrostatic diameter *D*_AD_ ≈ 300 nm; Supplementary Information), with its density tuned near the highly resistive *ν* = 0 state via a negative voltage applied to the bottom graphite gate. Lithographically etched line cuts in the top graphite define the TG and two side gates (SGs), as shown in Fig. 1b. The TG sets the filling factor in the bulk *ν*. Figure 1c shows the Landau fan outside the AD. A negative SG voltage controls the number of edge states transmitted through the constriction, which is formed by the ensemble of SGs and the AD.

## Coulomb oscillations at integer filling factors

We begin by studying a first device (AD 1) at a perpendicular magnetic field *B* = 5 T at integer bulk filling factors, *ν* = 1–4. By tuning the SG voltages, the innermost edge state can be brought into proximity with the AD-bound states. Figure 1d shows the diagonal conductance *G*_d_ as a function of the SG voltage *V*_sg_ at *ν* = 2.

When the constriction is set on a quantum Hall plateau, charge transport between the counterpropagating edges is suppressed, resulting in constant *G*_d_. However, when the inner extended edge is brought into proximity to the AD-bound states, charge carriers can tunnel from the extended edges to the AD. When the chemical potential of an AD-bound state aligns with the Fermi level, a quasiparticle can tunnel resonantly through the AD, leading to a dip in the tunnelling conductance *G*_d_. As the magnetic field is adiabatically varied by a small amount (by a fraction of the quantum flux), the AD-bound states adjust to maintain a constant enclosed flux. As the edge states move over the AD potential to adjust their size, their energy changes accordingly. This process generates periodic oscillations in *G*_d_ with a magnetic field period ${\Delta }B={\phi }_{0}/NA$, where *ϕ*_0_ is the quantum of magnetic flux, *N* is the number of tunnelling quasiparticles per quantum flux (*N* = *ν*_int_ in the IQH regime) and $A=\pi {D}_{{{\rm{AD}}}}^{2}/4$ is the AD area^38^.

The oscillations can also be driven electrostatically by varying the electron density via the TG or BG at constant area, giving rise to periodic tunnelling resonances. This allows direct measurement of the tunnelling charge *q* using ${\Delta{V}} = {q}/{CA}$, where *C* is the gate capacitance per unit area^24,36,37^. In the following, we varied the density using only the TG (for BG oscillations or area variations, see Supplementary Information).

**Fig. 2:**
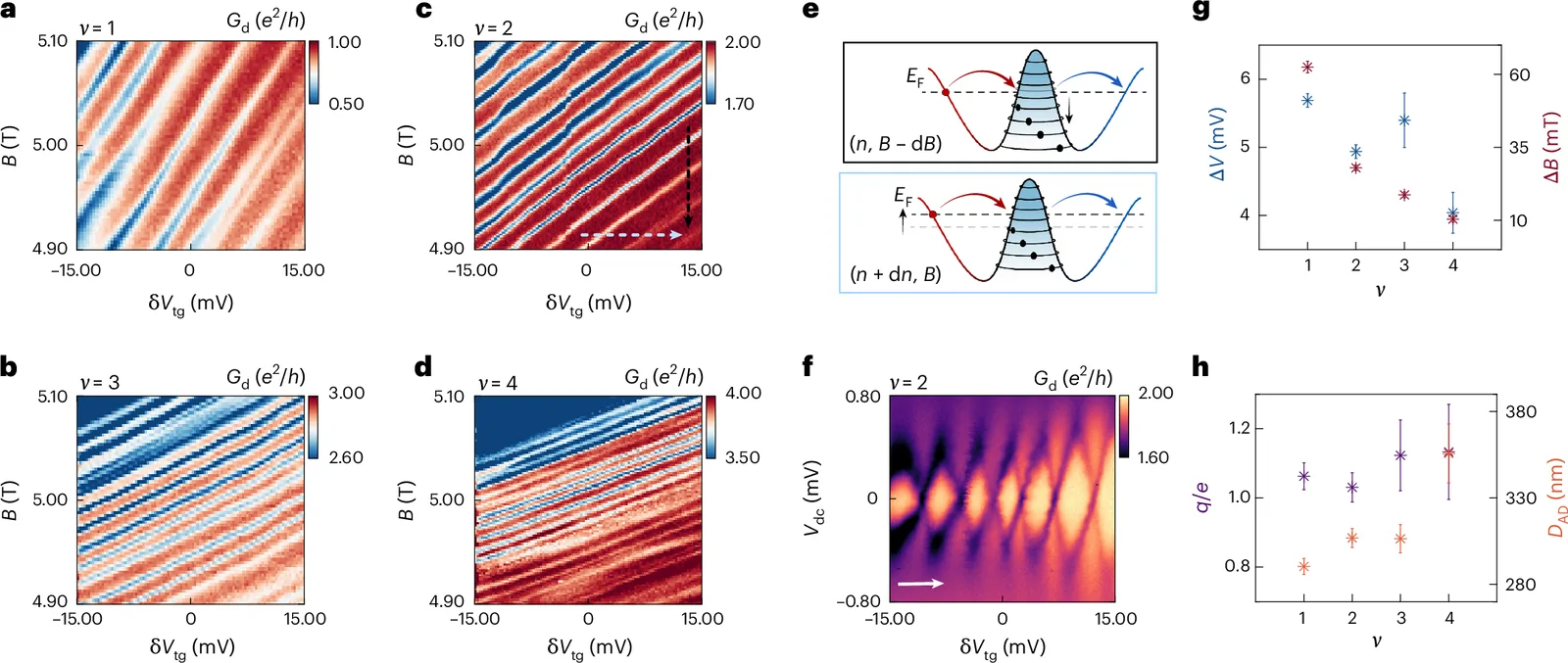
Coulomb-dominated oscillations for integer filling factors. **a**–**d**, Diagonal conductance *G*_d_ oscillations as a function of a variation of the TG voltage δ*V*_tg_ and the magnetic field *B* for the inner edge for filling factors *ν* = 1 (**a**), 2 (**c**), 3 (**b**) and 4 (**d**). **e**, Schematic of the oscillation mechanism when varying the magnetic field (top) or the charge carrier density (bottom), corresponding, respectively, to the black and light blue arrows in **c**. *E*_F_ is the Fermi energy. **f**, Diagonal conductance *G*_d_ as a function of a variation of the TG voltage δ*V*_tg_ and d.c. bias *V*_dc_, displaying characteristic Coulomb diamonds (trapezoidal regions where tunnelling through the AD is suppressed) for *ν* = 2 at *B* = 5 T. The white arrow indicates the direction of increasing number of electrons bound to the AD. **g**, TG period Δ*V* (blue) and magnetic field period Δ*B* (red) as functions of the filling factor *ν*. **h**, Measured tunnelling charge (purple) and AD diameter (orange) as functions of the filling factor *ν*. The error bars in **g** and **h** were determined by combining the procedure described in Methods with quadrature propagation of statistical errors.

For each filling factor, the SG voltages were set such that the transmission *t* ≈ 1. Figure 2a–d shows oscillations in the diagonal conductance as a function of the variation of the TG voltage and the magnetic field for the inner edge for filling factors *ν* = 1–4. A dip in the conductance appeared each time carriers could transfer from the higher to the lower potential edge through the AD-bound states, as illustrated in the schematic in Fig. 2e. To determine the oscillation period, we performed a two-dimensional fast Fourier transform (2D-FFT) (Supplementary Information). We extracted from the main peak frequency the magnetic field period Δ*B* and the TG period Δ*V*. The corresponding uncertainties were obtained following the method described in Methods. Figure 2g reports the extracted periods. Δ*V* slightly decreased with filling factor due to the increasing number of encircling edge states^33^, whereas Δ*B* decreased monotonically, consistent with the *ϕ*_0_/*N* dependence^18,38^. The latter yielded the AD diameter ${D}_{\mathrm{AD}}=2\sqrt{{\phi }_{0}/{\pi \Delta }B\,N}$, reported in orange in Fig. 2h, which increased with the filling factor. Using the geometric capacitance of the TG, *C*, and substituting the area dependence from the magnetic field and gate period, the charge of the tunnelling particle can be written as

1 $$q=\frac{C{\phi }_{0}\Delta {{V}}}{N\Delta {{B}}}.$$

Figure 2h shows the computed charge (purple) for different filling factors. The calculated charge was 5% higher than the expected value of one electron per period. We attribute this discrepancy to a small dependence of the AD potential on the TG voltage, as reported in Supplementary Information. Finally, we studied the d.c. bias V_dc_ dependence of the oscillations. Figure 2f shows that the diagonal conductance *G*_d_ exhibited Coulomb diamond behaviour.

## Coulomb oscillations at fractional filling factors

**Fig. 3:**
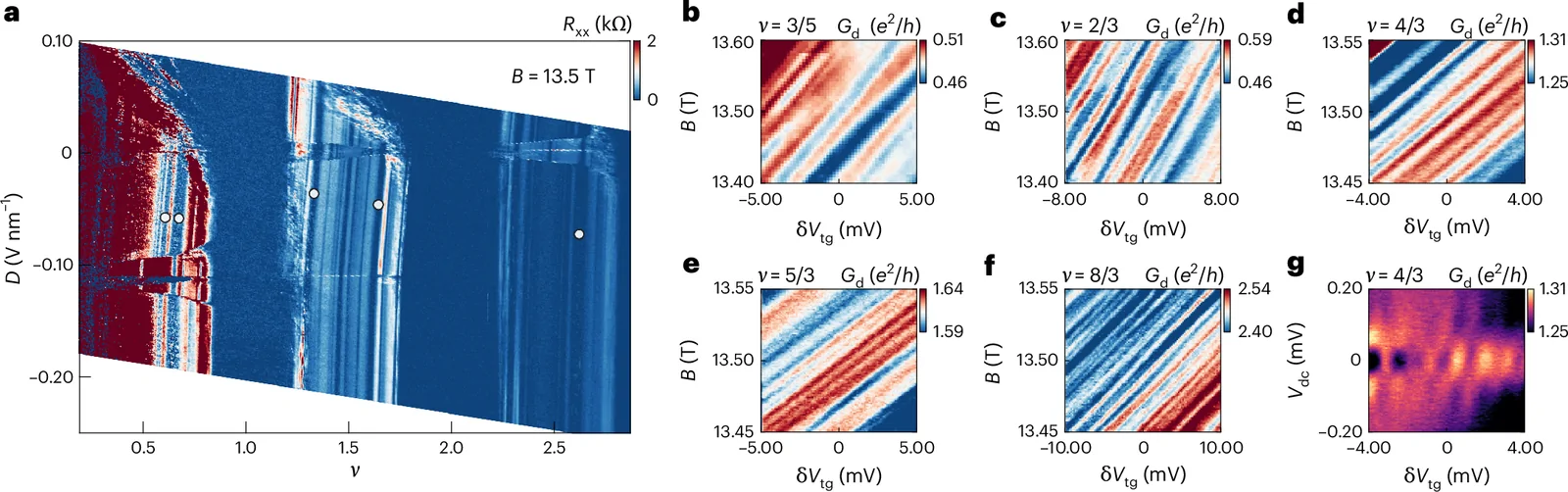
Oscillations in the FQH regime. **a**, Longitudinal resistance *R*_xx_ measured outside the AD as a function of the bulk filling factor *ν* and the displacement field *D* at *B* = 13.5 T. This map closely resembles similar results observed in bilayer-graphene, and it shows the emergence of strong even-denominator states, *ν* = 1/2, 3/2 and 5/2 in the first orbital LLs^50–54^. However, due to a highly resistive charge neutrality point, it was not possible to observe any states below *ν* = 1/2, such as *ν* = 1/3 and 2/5. **b**–**f**, Diagonal conductance oscillations *G*_d_ as a function of the variation of the TG voltage δ*V*_tg_ and the magnetic field *B* for *ν* = 3/5 (**b**), 2/3 (**c**), 4/3 (**d**), 5/3 (**e**) and 8/3 (**f**). The white dots in **a** indicate the values at which the oscillations were taken. **g**, Diagonal conductance oscillations *G*_d_ as a function of the variation of the TG voltage δ*V*_tg_ and the d.c. bias *V*_dc_ for *ν* = 4/3.

Next, we turn to the FQH regime. For this part of the study, we fabricated a second device (AD 2) with a richer set of FQH states. Figure 3a shows the longitudinal resistance *R*_xx_ measured outside the AD as a function of the bulk filling factor *ν* and the displacement field *D* at *B* = 13.5 T.

For each fractional filling factor, we repeated the procedure outlined for the integer case. The back gate was set to *ν* ≤ 0, and the displacement field *D* was kept close to zero to ensure that all states were measured under comparable conditions. Figure 3b–f shows the diagonal conductance oscillations observed at fractional filling factors *ν* = 3/5, 2/3, 4/3, 5/3 and 8/3. The displacement field *D* and the corresponding *ν* values at which these oscillations were observed are marked by white dots in Fig. 3a.

For all oscillations, we performed a 2D-FFT to extract the oscillation periods following the same procedure used for the IQH regime described in Methods, as well as the d.c.-bias dependence, as shown in Fig. 3g for *ν* = 4/3.

**Fig. 4:**
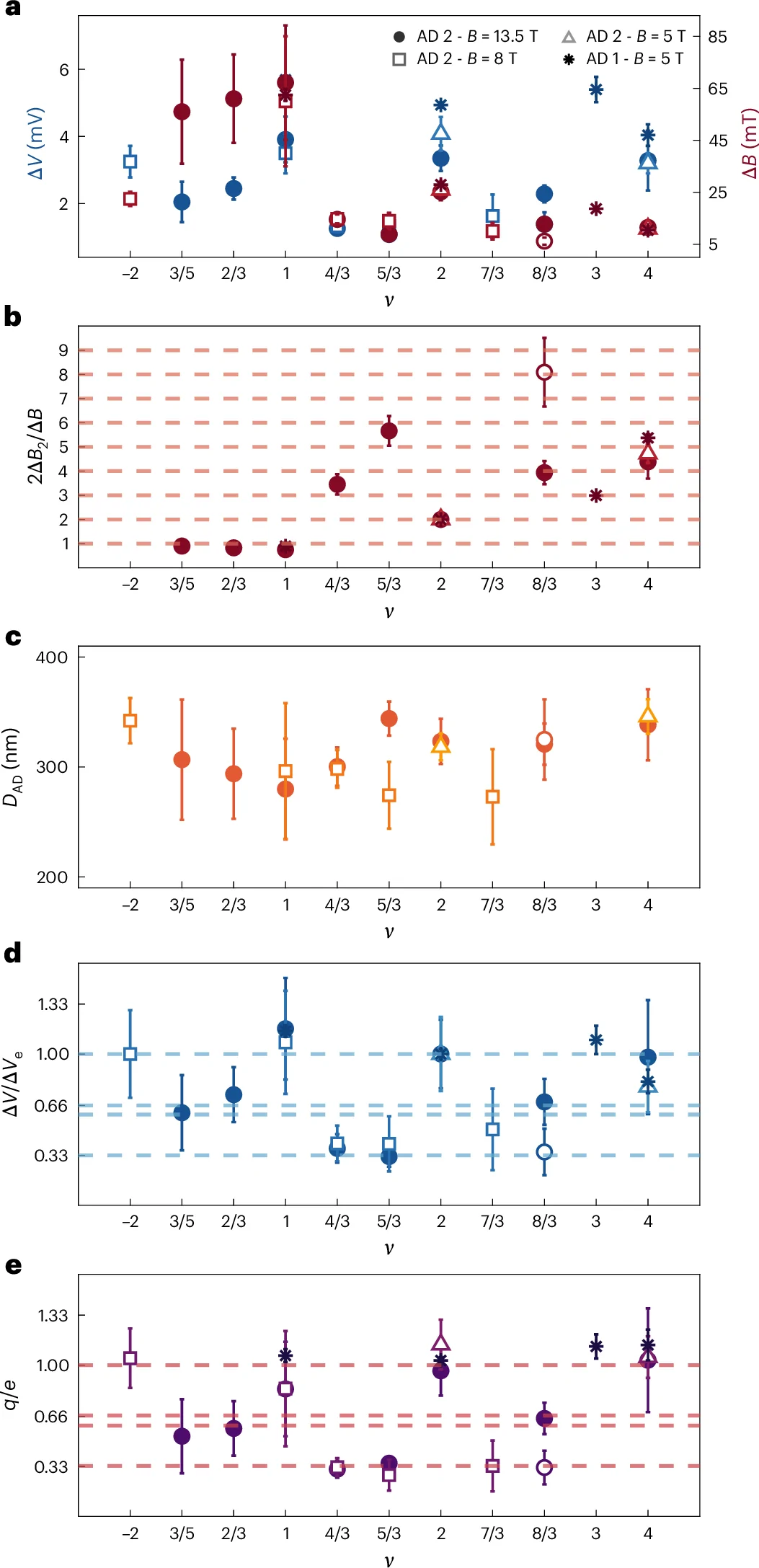
Charge measurement. **a**, TG period Δ*V* (blue) and magnetic field period Δ*B* (red) as functions of the filling factor *ν* for oscillations measured at different magnetic fields and for two devices: AD 1 for integer filling factors and AD 2 for both integer and fractional filling factors. **b**, Ratio of the field period at *ν* = 2, Δ*B*_2_, to the field period at each filling factor. The factor of 2 accounts for the *ϕ*_0_/2 periodicity characteristic of *ν* = 2. **c**, AD diameter as a function of filling factor *ν*. **d**, Ratio of the gate period at each filling factor to the gate period corresponding to a single electron, Δ*V*_e_. For AD 2 at *B* = 8 T, Δ*V*_e_ = Δ*V*_−2_ was used, whereas for all other cases Δ*V*_e_ = Δ*V*_2_. **e**, Quasiparticle charge as a function of filling factor, computed using equation (1). In all panels, the empty circle indicates the smaller oscillation at *ν* = 8/3. In **b**, the data for AD 2 at *B* = 8 T are not reported, as measurements at *ν* = 2 were not performed. In **c**, data for AD 1 are omitted for clarity as they correspond to a different device. The error bars were determined by combining the procedure described in Methods with quadrature propagation of statistical errors.

Figure 4a shows the extracted gate periods (red) and field periods (blue) for various filling factors, measured for the two different devices (integer filling factors in ADs 1 and 2 and fractional filling factors in AD 2) and at different magnetic fields. Although this section focuses on the fractional states, we have also included the integer states for completeness and clarity. For *ν* = 8/3, measured at a magnetic field *B* = 13.5 T, we report two data points (full and empty circles), corresponding to the two observed oscillations described in Supplementary Information. The smaller oscillation has almost half the period of the larger data point, corresponding to an oscillation appearing between two larger oscillations. However, as this occurred only in certain regions, we decided to retain both points. In the following, we refer to them as the small (empty circle) and large (full circle) 8/3 oscillations, according to their respective periods.

A decrease in the magnetic field period with increasing filling factor was observed, consistent with a *ϕ*_0_/*N* dependence. The constant *N*, determined solely by the filling factor of the interacting edge, is the number of quasiparticles with charge *q* transferred upon an increase of the magnetic flux by *ϕ*_0_ (ref. ^38^). We extracted these values by comparing the magnetic field periods. Assuming a constant area, the ratio of two field periods is inversely proportional to the ratio of their *N* values, namely $\Delta {B}_{\nu }/\Delta {B}_{{\nu }{{\prime} }}={N}_{{\nu }{{\prime} }}/{N}_{\nu }$. We carried out this comparison using the oscillations obtained at filling factor *ν* = 2 at their respective magnetic fields. The ratio 2Δ*B*_2_/Δ*B* = *N* is plotted in Fig. 4b. The oscillations for AD 2 at *B* = 8 T are not reported, as we did not perform measurements at *ν* = 2. The results for filling factors *ν* = 3/5, 2/3 and 1 closely follow the *N* = 1 line, consistent with our integer results as well as with previous studies^27,37^. Moreover, the results for filling factors *ν* = 4/3 and 5/3 are close to *N* = 4 and *N* = 5, respectively. We observed that the large *ν* = 8/3 oscillation was close to *N* = 4, whereas the small oscillation was close to *N* = 8, the latter agreeing with its expected value corresponding to a tunnelling charge equal to the fundamental charge for the filling factor, *q* = e^*^ = 1/3. For *ν* = 3, the integer result was recovered. However, for *ν* = 4, the observed value exceeded the expected value of *N* = 4, which can be attributed to a variation of the area, as will be discussed below.

Next, using the previously determined values of *N*, we computed the AD diameter *D*_AD_ for different filling factors, as shown in Fig. 4c. All diameters clustered around the same value *D*_AD_ ≈ 300 nm, consistent with the correct estimation of *N*.

The ratio between the gate periods provides information about the charge of the tunnelling quasiparticle. Assuming a constant area across different filling factors, the relation $\Delta {V}_{\nu }/\Delta {V}_{{\nu }{{\prime} }}={q}_{\nu }/{q}_{{\nu }{{\prime} }}$ holds, where *q*_*ν*_ is the charge of the tunnelling quasiparticle at filling factor *ν*. Using the integer filling factors as a reference for the electron charge, this can be expressed as Δ*V*/Δ*V*_e_ = *q*/*e*, where Δ*V*_e_ is the gate voltage required to tunnel a single electron. Figure 4d shows this ratio for different filling factors. For AD 2 at *B* = 8 T, Δ*V*_e_ = Δ*V*_1_ was used, whereas for all other cases, Δ*V*_e_ = Δ*V*_2_. As expected for the IQH regime, filling factors *ν* = −2, 1, 2, 3 and 4 consistently fell around the *q*/*e* = 1 line, independently of the magnetic field at which they were measured. The filling factor *ν* = 3/5 appeared close to *q*/*e* = 3/5, which is a deviation from the expected value of *q*/*e* = 1/5 (ref. ^39^). Filling factors *ν* = 2/3 and the large 8/3 oscillation followed the *q*/*e* = 0.66 line, as expected for *ν* = 2/3 and in agreement with previous results for GaAs ADs^27^. Finally, the filling factors *ν* = 4/3, 5/3, 7/3 and the small 8/3 clustered close to *q*/*e* = 0.33, with *ν* = 7/3 showing a larger uncertainty due to the difficulty in identifying the oscillation period.

Finally, we used equation (1) to compute the charge of the tunnelling quasiparticles for each filling factor, employing the same values of *N* used to determine the AD diameter. Figure 4e shows the quasiparticle charge as a function of filling factor. The values align with those obtained from the gate-period ratios in Fig. 4d, confirming that the assumption of a nearly constant area across filling factors is reasonable. For integer filling factors, *q*/*e* was close to 1, as expected for tunnelling of electrons. Fractional filling factors *ν* = 4/3, 5/3, 7/3 and the small 8/3 oscillation fell near *q*/*e* = 0.33, whereas *ν* = 2/3 and the large 8/3 oscillation were close to *q*/*e* = 0.66. Finally, *ν* = 3/5 appeared near *q*/*e* = 3/5.

## Discussion

**Fig. 5:**
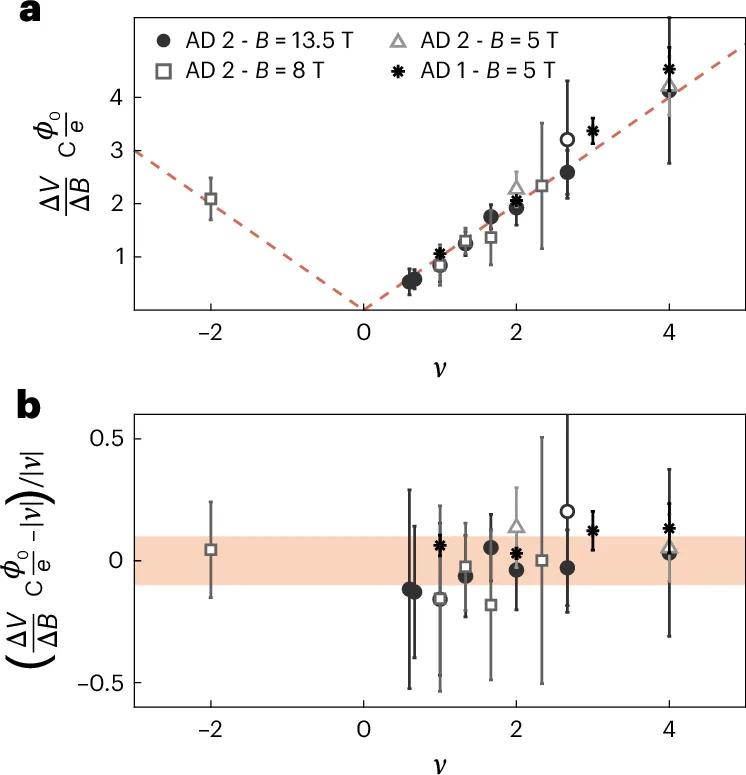
Oscillation slope. **a**, Ratio of the TG period to the magnetic field period, normalized by the TG capacitance per unit area and *ϕ*_0_/*e*, as a function of the filling factor *ν*. The dashed orange lines are guides to the eye. The oscillations exhibit a slope corresponding to the filling factor *ν*. **b**, Relative error of the ratio of the TG period to the magnetic field period minus the filling factor. The shaded orange band marks the ±10% interval. The error bars were determined by combining the procedure described in Methods with quadrature propagation of statistical errors.

Using the definition of the filling factor *ν* = *nϕ*_0_/*B* and considering small variations Δ*n* (induced by Δ*V*) and Δ*B*, we note that the oscillation slope $\frac{C{\phi }_{0}\Delta V}{eN\Delta B}$ always followed the filling factor *ν* at which they oscillations are measured, as shown in Fig. 5a. Consequently, once *N* was determined, the ratio Δ*V*/Δ*B* always yielded the fundamental charge e^*^:

2 $$\frac{q}{e}=\frac{C{\phi }_{0}\Delta V}{eN\Delta B}=\frac{\nu }{N}.$$

This can lead to a misinterpretation, with the extracted value being ascribed to the tunnelling quasiparticle charge rather than to the slope of the oscillations determined by the filling factor^24^. This is why it is essential to compare the value obtained from the charge equation (equation (1)) with the ratio Δ*V*/Δ*V*_e_, which provides a cross-check for the correct charge determination. In our case, Fig. 4d,e shows agreement among all measured filling factors, supporting the validity of this approach.

Moreover, as the plotted ratio accounts only for density variations (Δ*n* = *C*Δ*V*/*e*), deviations from the orange line indicate changes in the enclosed area. As shown in Fig. 5b, most data points lie within the ±10% band, confirming that area variations remained small, with a larger discrepancy appearing only at higher filling factors, such as *ν* = 4.

As the robustness of the analysis has now been demonstrated, we can proceed to discuss the measured tunnelling charges. We observed that for *ν* = 4/3, 5/3, 7/3 and the small 8/3 oscillation, the measured quasiparticle charge *q*/*e* was close to the expected value of the minimal excitation e^*^ = 1/3. However, for *ν* = 2/3 and the large *ν* = 8/3, the measured values were closer to *q*/*e* = 2/3. This is consistent with the only previous AD measurement at *ν* = 2/3 in GaAs (ref. ^27^). Note that shot noise measurements in GaAs found the charge at *ν* = 2/3 to be *q*/*e* = 2/3 at low temperature as well (but *q*/*e* = 1/3 at higher temperatures)^11,40^. Although experiments that simultaneously measured both auto- and cross-correlation ruled out quasiparticle bunching as the underlying mechanism^41^, no detailed explanation has yet been given for the occasional observation of a *q* = 2/3 charge.

The naive picture of the 2/3 edge consists of a downstream conductance-1 mode and an upstream 1/3 mode. However, Kane, Fisher and Polchinski showed that in the presence of disorder-induced scattering between the edges, the structure renormalizes to a single downstream mode carrying conductance 2/3 with an upstream neutral mode^42,43^. Although at short length scales and high temperatures one might see the first structure, at the lowest temperatures and longest length scales, one should only see the renormalized structure.

Pursuing this line of reasoning, we repeated the renormalization group calculation with *n* > 1 downstream unit-conductance modes and an upstream conductance-1/3 mode (Supplementary Information). We found that under most conditions, the equilibrated system did not include a mode carrying 2/3 conductance at the lowest temperature but instead included a 1/3 mode. However, in a scenario where the *n* integer modes were physically separated from the 2/3 edge, such that the 2/3 renormalized by itself, we rediscovered the Kane–Fisher–Polchinski result.

We argue that this scenario is potentially in agreement with our experiments as well as with previous works^11,27,40^. In fact, because the *ν* = 1 charge gap is fairly small, the edge modes were not physically well separated from the nearby fractional edges. Thus, the *ν* = 5/3 edge should be in a regime where we observe a conductance-1/3 mode, in agreement with our observations. By contrast, the larger *ν* = 2 charge gap suggests that the fractional *ν* = 8/3 edge can maintain a conductance-2/3 mode over a broad temperature range, again matching the observed *q* = 2/3 charge.

We emphasize that this renormalization group calculation is not meant to be a full calculation of the expected AD oscillations, but it does strongly point towards a direction for future theoretical analysis. Furthermore, we note that the presence of a conductance-2/3 mode at the fixed point does not immediately imply a tunnelling charge *q*/*e* = 2/3; it merely allows for that possibility. For example, at the Kane–Fisher–Polchinski fixed point, both *q*/*e* = 1/3 and *q*/*e* = 2/3 have the same scaling dimension.

However, we should also not neglect the possibility that Coulomb charging effects, as described by Rosenow and Halperin^44,45^, played an important role. In particular, for two oscillations with different tunnelling amplitudes, if one of them dominates over the other, we would be able to detect only the strongest one, resulting in an apparent doubling of the tunnelling charge.

The physics at *ν* = 3/5 is like that at *ν* = 2/3, in that the state also renormalizes to a single mode carrying conductance 3/5 and several neutral modes. Shot noise measurements have similarly shown that at low temperatures, the measured Fano factor is closer to 3/5, like our measured tunnelling charge, whereas at higher temperatures, it approaches the expected value of *e*/5 (ref. ^13^).

## Conclusion

In conclusion, we have demonstrated that a gate-defined bilayer-graphene AD serves as a powerful yet simple platform for measuring fractional charge in the quantum Hall regime. Owing to its versatile design, this platform can be readily extended to other material systems, for instance, for charge measurements in anomalous quantum Hall states in rhombohedral graphene^46,47^ or in twisted transition-metal dichalcogenides^48,49^. Moreover, our results indicate that the number of integer edge modes in hole-conjugate states may govern edge equilibration according to their parity. These findings highlight the critical importance of understanding the re-equilibration processes as a prerequisite for accessing and controlling non-abelian quantum Hall phases.

## Methods

### Sample fabrication

The devices were fabricated using a standard van der Waals dry-transfer technique. First, graphene and hBN were mechanically exfoliated from bulk crystals on an SiO_2_/Si substrate. The desired flakes were identified under an optical microscope and an atomic force microscope to check for any flake impurities. The stack was assembled using home-made poly(bisphenol A carbonate) (PC)/polydimethylsiloxane to pick up all the flakes at a temperature of approximately 90 °C. The stack was then transferred onto a doped silicon substrate with a 285-nm-thick layer of thermally grown SiO_2_ by melting the PC at a temperature of approximately 180 °C. The measured stacks for AD 1 (AD 2) had a 56-nm (45-nm) top hBN layer and a 33-nm (30-nm) bottom hBN layer.

Devices were patterned through several steps of electron-beam lithography followed by reactive-ion etching and metal deposition. First, the TG contacts were created by depositing an 18-nm Pd layer. For AD 1, the device geometry was then defined through reactive-ion etching, initially using O_2_, followed by SF_6_ and then another O_2_ step. The electrode pattern was subsequently created by etching with SF_6_ and O_2_, followed by angled deposition of a 5 nm/100 nm Cr/Au layer with rotation. For AD 2 instead, the device geometry was defined through reactive-ion etching, initially using O_2_ and followed by CHF_3_ + O_2_. The electrode pattern was subsequently created by etching with O_2_ and then CHF_3_ + O_2_, followed by angled deposition of a 5 nm/12 nm/60 nm Cr/Pd/Au layer with rotation.

Finally, the TG was refined in 30-s O_2_ etch steps. During each step, the two-probe resistance between each gate bridge-contact was monitored to ensure that all gates were fully separated while minimizing etching of the hBN and achieving narrow line widths^55^.

### Measurements

Devices were measured in a dry dilution refrigerator (BlueFors) with a base temperature of approximately 10 mK. Electronic filters were installed on all transport lines to thermalize electrons. Resistances were measured with lock-in amplifiers (MFLI, Zurich Instruments) with a 1-V a.c. voltage excitation at 33.333 Hz for AD 1 and 107.777 Hz for AD 2, applied through a home-made voltage divider. The current was measured through a K-tip variable-gain transimpedance amplifier, and voltages were amplified by 100 at room temperature using low-noise voltage preamplifiers (LI-75A) before being sent to the lock-in amplifier. The d.c. voltage bias was set by a Yokogawa GS200, and for d.c. bias measurements, we employed a home-made voltage adder. Gate voltages were applied independently to each gate using dedicated sources (GS200, Yokogawa). To improve the ohmic contact quality, a positive silicon gate voltage *V*_Si_ = 14.5 V for AD 1 and *V*_Si_ = 53.2 V for AD 2 was applied, which drove the graphene layer adjacent to the contacts into a highly electron-doped regime.

The devices had ohmic contacts along the path of both upstream and downstream extended edges, allowing for current injection and differential chemical potential measurements across the AD. Unless otherwise stated, all measurements were performed with an applied voltage *V*_ac_ = 10 μV (3 μV) for the IQH (FQH) regime, while we measured the transmitted current *I*_t_. The longitudinal resistance *R*_xx_ = *V*_xx_/*I*_t_ was measured outside the AD, and the transmitted resistance *R*_t_ = *V*_t_/*I*_t_ was measured across it. Measuring the diagonal voltage drop gave the diagonal conductance through the AD, *G*_d_ = *I*_t_/(*V*_d_) ≡ *ν*_c_*e*^2^/*h*, where *ν*_c_ is the constriction filling factor.

### Period and error estimation of the oscillation

For each filling factor, the 2D-FFT provides information about the periodicity of the oscillations. However, the 2D-FFT can produce artefacts because the oscillations are not infinite but are abruptly truncated at the limits of the magnetic field range and gate voltage range and due to the presence of a background. To reduce these artefacts, a linear background is subtracted from the raw data before performing the 2D-FFT to remove low-frequency features. Additionally, a windowing function was applied to the curves before performing the 2D-FFT:

$$\tilde{S}(\,{f}_{{{\rm{B}}}},{f}_{{{\rm{V}}}})={{{\mathcal{F}}}}_{2D}\left[W(B,V\,)\times S(B,V\,)\right]$$

where *S*(*B*, *V*) is the signal and *W*(*B*, *V*) is the windowing function, in our case the Hamming function with the window size equal to the full gate and field range.

The characteristic period was typically extracted from the highest peak of the 2D-FFT. In some cases, however, particularly for certain regimes in the FQH regime, one peak could not be unambiguously identified. In such cases, the periodicity was, instead, estimated from the spacing between consecutive oscillations directly in the original conductance signal. The oscillations selected were at the centre of the conductance map, both along the magnetic field axis (horizontal) and along the gate voltage axis (vertical). This choice was motivated by the finite extent of the plateau, which typically spanned only approximately 200 mT and 50 mV. The choice can affect the periodicity of the oscillations near its boundaries. Furthermore, the TG simultaneously controls the coupling between the AD and the extended edge states, introducing another source of variation in the oscillation periodicity. This non-uniformity is one of the reasons why the 2D-FFT did not always yield sharp, well-resolved peaks, but instead produced broader spectral features.

**Extended Data Fig. 1:**
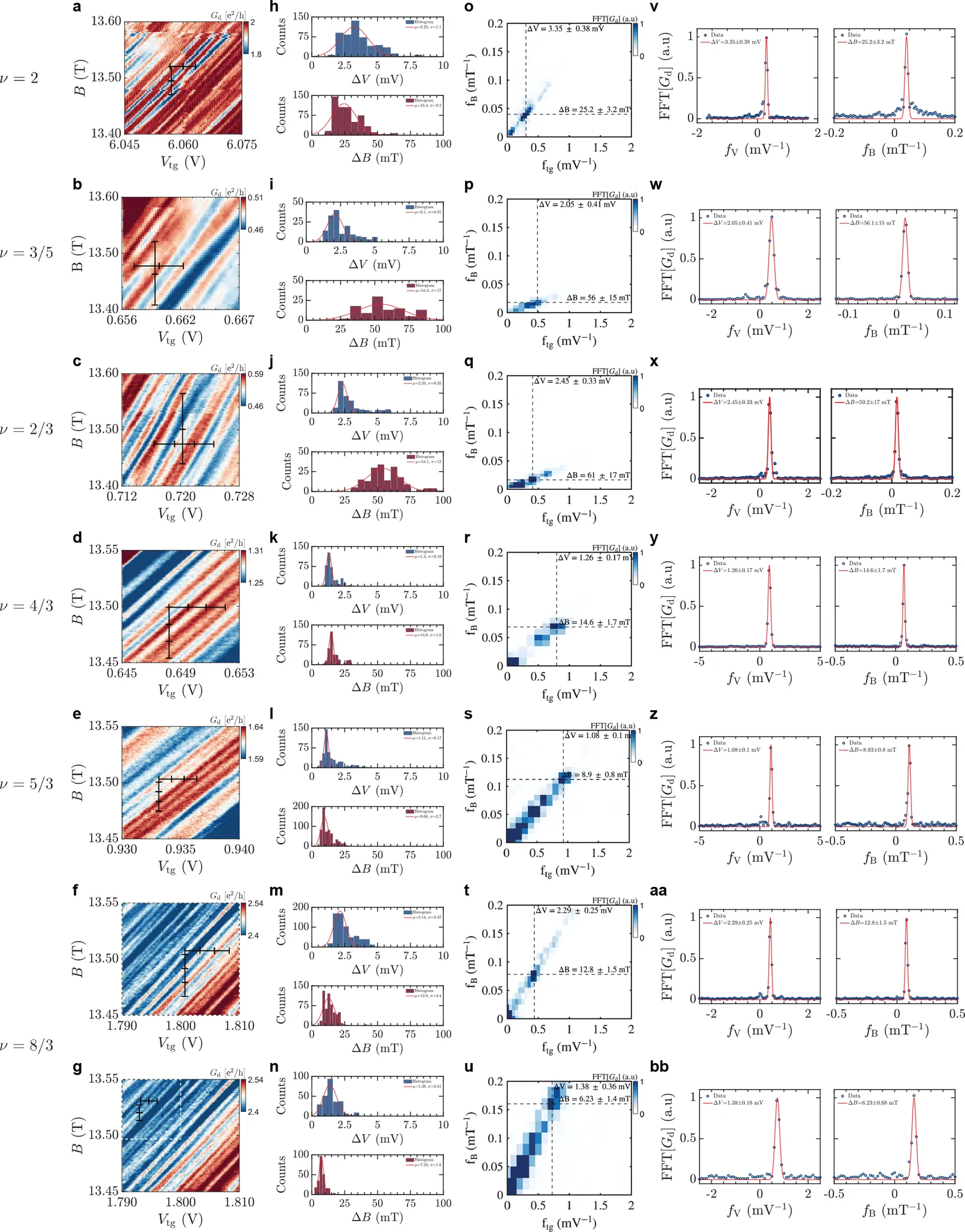
Period and uncertainty estimation at 13.5 T. Procedure to extract the correct periodicity and its uncertainty for all fractional states and the reference integer state *ν* = 2 used in the main text at 13.5 T. **a-g**, Diagonal conductance as a function of top gate voltage and magnetic field. The black lines indicate the peaks used as a first estimate of the period. **h-n**, Histograms obtained by extracting the period from each top gate voltage and magnetic field line of the conductance maps. The data are fitted with a Gaussian to extract the mean value and its uncertainty. **o-u**, 2D-FFT of the oscillations. **v-bb**, Gaussian fit of the 2D-FFT peaks used to extract the periodicity and its associated uncertainty.

**Extended Data Fig. 2:**
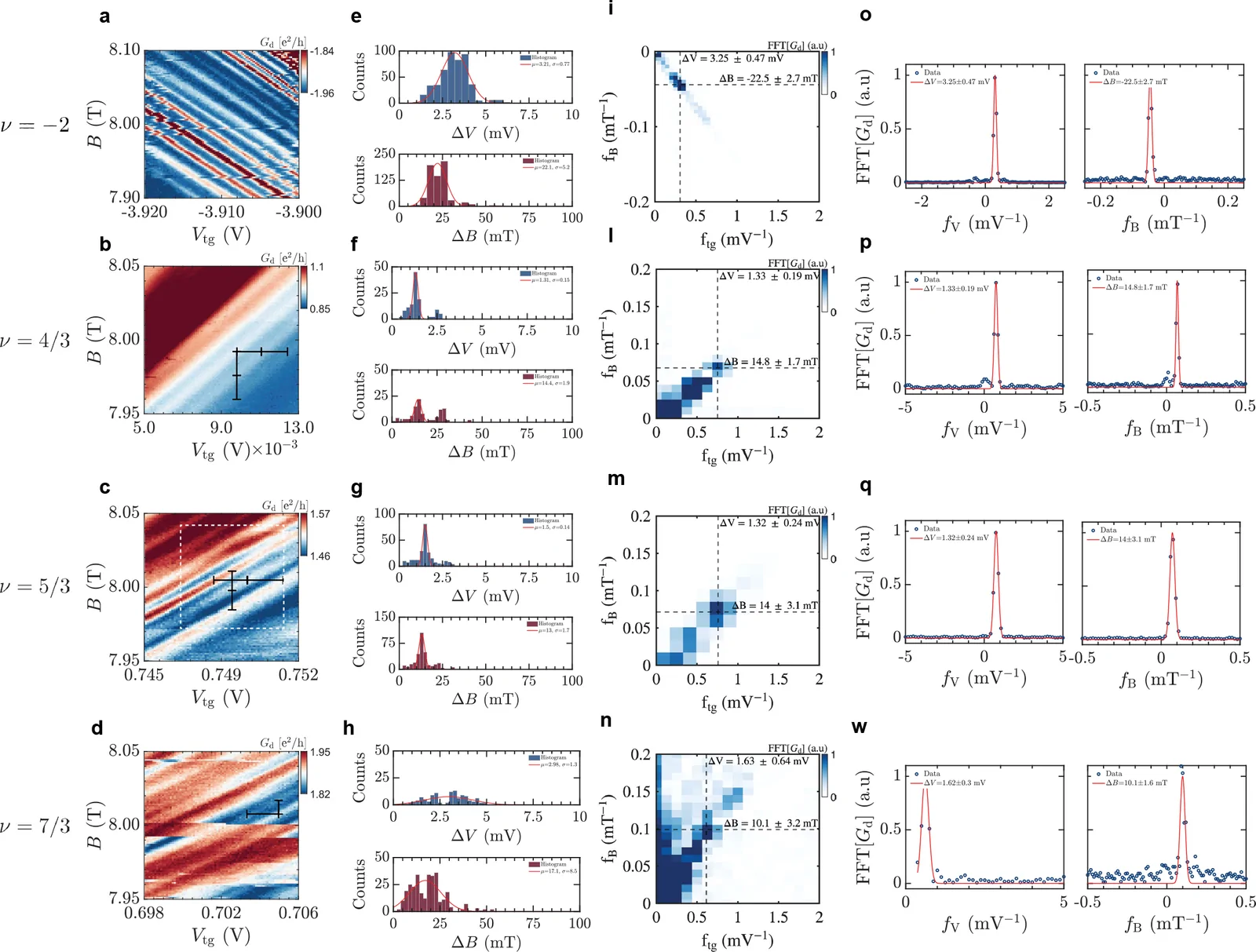
Period and uncertainty estimation at 8 T. Procedure to extract the correct periodicity and its uncertainty for all fractional states and the reference integer state *ν* = − 2 used in the main text at 8 T. **a-d**, Diagonal conductance as a function of top gate voltage and magnetic field. The black lines indicate the peaks used as a first estimate of the period. **e-h**, Histograms obtained by extracting the period from each top gate voltage and magnetic field line of the conductance maps. The data are fitted with a Gaussian to extract the mean value and its uncertainty. **i,l-n**, 2D-FFT of the oscillations. **o-q,w**, Gaussian fit of the 2D-FFT peaks used to extract the periodicity and its associated uncertainty.

To extract the period in a systematic and consistent manner, we adopted the following procedure whenever no clearly identifiable peak was present in the 2D-FFT. If a distinct peak was found that yielded a value consistent with the periodicity observed in the conductance map, that value was used directly. Otherwise, the procedure described below was applied. Extended Data Figs. 1 and 2 report the full procedure for all fractional oscillations used in the main text at *B* = 13.5 T and *B* = 8 T, respectively.

1. We first visually identified the relevant oscillation period from the two-dimensional conductance map. For each dataset, the oscillations used for period identification are explicitly marked. We preferentially selected oscillations near the centre of the gate voltage and magnetic field range.
2. For each value of TG voltage and magnetic field, we identified peaks in the oscillations using the MATLAB findpeaks function. The period was then estimated from the pairwise differences between all detected peaks, and the resulting values were compiled into a histogram (Extended Data Figs. 1h–n and 2e–h). The bin width was set to twice the measurement step size, reflecting the minimum systematic uncertainty of the measurement set-up.
3. The histogram was fitted with a Gaussian function to extract the mean value.
4. Using the mean period obtained in step 3, we identified the corresponding peak in the 2D-FFT closest to that value (Extended Data Figs. 1o–u and 2i–n).
5. A line cut through the 2D-FFT at the identified peak frequency was fitted with a Gaussian. The mean and variance of this fit were used as the final estimates of the period and its uncertainty, respectively (Extended Data Figs. 1v–bb and 2o–w).

The error obtained in this way accounts for both the measurement uncertainties and the statistical uncertainty of the oscillations.

Most of the measurements presented in the main text were carried out with a discrete magnetic field step of d*B* = 1 mT, the lower limit of our magnet power supply, and a gate voltage step of d*V* = 0.1 mV, with scans containing approximately *N*_B_ = *N*_V_ = 101 points. The minimal resolvable frequency in the FFT is given by the range of the oscillations:

$$\delta {f}_{{\rm{V}}}\approx\frac{1}{{N}_{{\rm{V}}}\,{\rm{d}}V},\qquad \delta {f}_{{\rm{B}}}\approx\frac{1}{{N}_{{\rm{B}}}\,{\rm{d}}B}.$$

However, the largest source of uncertainty arises from the finite size of the FQH plateaux, which typically extended over a limited range. This meant that only a limited number of oscillations could be observed in both the magnetic field and gate voltage before the system left the fractional plateau. In practice, no more than about ten oscillations can be resolved; after this point, the bulk was no longer in the FQH plateau, making it difficult to extract the periodicity with high precision. This limitation directly increased the error on the extracted frequency, as the frequency resolution is inversely proportional to the available range. Thus, the shorter plateau length in the FQH regime compared with the IQH regime is the dominant factor that limited the precision of the periodicity analysis.

For *ν* = 8/3 at *B* = 13.5 T, as described in Supplementary Information, we performed the 2D-FFT in two separate regions to correctly identify the periodicity. For the small oscillations, we took for the error on the period twice the statistical uncertainty to account for the difficulty in identifying the peak, given the smaller number of observable oscillations.

For *ν* = 7/3 at *B* = 8 T, the histogram had a very wide distribution, which we attribute to the low visibility of the oscillations and the consequent difficulty in reliably identifying peaks with findpeaks. In this case, we relied solely on the periods indicated by the lines drawn on the plot and applied the procedure from step 4 onwards. Our prediction was supported by the d.c. bias map, which shows diamonds with a similar period to that estimated. We adopted for the error a conservative estimate of twice this value to reflect the higher uncertainty in this measurement.

The MATLAB codes used for the period and error estimation are available via Zenodo^56^.

### Interpretation of *N*

Previous AD studies have focused only on the IQH effect or on the FQH effect at filling factors below *ν* = 1 (refs. ^21,24,26,27,36–38,57,58^), where *N* = 1 (for example, for *ν* = 2/3), or for integer filling factors, *N* = *ν*. However, *N* should be understood as the number of quasiparticles transferred per quantum flux *ϕ*_0_. This interpretation is particularly clear for FQH states in a higher LL, where *N* takes different values, for example, *N* = 4 at *ν* = 4/3 and *N* = 5 at *ν* = 5/3. The edge-counting approach used in some previous works^27^ would imply that there are only two edges for *ν* = 4/3, leading to an estimated AD diameter far larger than the lithographic size between the SGs (~450 nm).

## Online content

Any methods, additional references, Nature Portfolio reporting summaries, source data, extended data, supplementary information, acknowledgements, peer review information; details of author contributions and competing interests; and statements of data and code availability are available at https://doi.org/10.1038/s41567-026-03412-2.

## Supplementary information

Supplementary Information Supplementary Figs. 1–18 and Discussions (‘Devices characterization’, ‘Device tuning’, ‘Oscillations in the IQH regime’, ‘Oscillation in bottom gate’, ‘Oscillation in area’, ‘Outer edge at *ν* = 2’, ‘Oscillations in the FQH regime’ and ‘Renormalization group calculation’).

Peer Review file

## Data Availability

The data supporting the findings of this study are available via Zenodo at https://doi.org/10.5281/zenodo.20611205 (ref. ^56^). Other data are available from the corresponding author upon request.

## Code availability

The codes supporting the findings of this study are available via Zenodo at https://doi.org/10.5281/zenodo.20611205 (ref. ^56^).
