# Supplementary material for: Quantum Hall antidot as a fractional coulombmeter
Source: Nat Phys. 2026 Aug 14;22(10):1587–93. doi: 10.1038/s41567-026-03412-2 (PMC13658039; doi:10.1038/s41567-026-03412-2)
Supplement: Supplementary file 1 [file 41567_2026_3412_MOESM1_ESM.pdf]

nature physics

Supplementary information https://doi.org/10.1038/s41567-026-03412-2

Quantum Hall antidot as a fractional coulombmeter

In the format provided by the authors and unedited

SUPPLEMENTARY MATERIALS

CONTENTS

Supplementary Materials 1
A. Devices Characterization 2
B. Device Tuning 4
Silicon Gate Characterization 4
Side Gates Characterization 4
C. Oscillations in the IQH regime 5
AD #1 5
AD #2 7
D. Oscillation in Bottom Gate 9
E. Oscillation in Area 11
F. Outer Edge at $\nu = 2$ 12
G. Oscillations in the FQH regime 13
Oscillations at $\nu = 8/3$ 18
H. Renormalization Group Calculation 19

References 21

A. Devices Characterization

Figure S1a,e shows optical microscope images of the devices. The black dashed square marks the location of the AD. To ensure that ballistic edge channels encircle the AD, its diameter must be significantly larger than the magnetic length (l_{B}), $D_{\mathrm{AD}} \gg l_{\mathrm{B}}$. Figures S1b,f present AFM height sensor images of the AD #1 and AD #2. From the height sensor, we measure the etched AD diameters to be $D_{\mathrm{AD}\#1} = 190$ nm and $D_{\mathrm{AD}\#2} = 195$ nm. However, because of the electric field propagation through the thicker top hBN layer, the effective area of the AD at the graphene surface is expected to be larger. Using a top hBN thickness of approximately 50 nm, we estimate the effective AD diameter to be approximately 300 nm.

From the measurements Hall conductance dependence, measured outside the AD, we extract the BG capacitance per unit area $C^1_{\mathrm{bg}} = 605 \pm 2\ \mu\mathrm{Fm}^{-2}$ and from the slope in Fig. S1c the TG Capacitance $C^1_{\mathrm{tg}} = 451 \pm 5\ \mu\mathrm{Fm}^{-2}$ for the first device AD #1. While for the second device we measure $C^2_{\mathrm{bg}} = 805 \pm 2\ \mu\mathrm{Fm}^{-2}$ and from the slope in Fig. S1h the TG Capacitance $C^2_{\mathrm{tg}} = 549 \pm 5\ \mu\mathrm{Fm}^{-2}$.

Following the procedure in [1] the measured R_{xx} can be fitted with the following equation

$$R_{\mathrm{xx}} = \frac{L/W}{\mu\sqrt{(C_{\mathrm{bg}}(V_{\mathrm{bg}} - V_{\mathrm{cnp}}))^2 + (n_0 e)^2)}} \tag{1}$$

with V_{cnp} the voltage corresponding to the charge neutrality point, L and W the length between two contacts and the width of the device respectively, μ the mobility at high density, and n_0 the charge background fluctuations, which are proportional to the intrinsic doping. From the best fit, shown in Fig. S1d we find a mobility $\mu^1 \approx 180,000$ $\mathrm{cm}^{-2}\mathrm{V/s}$ and a charge impurity of $n^1_0 = 1.1 \cdot 10^{10}\ \mathrm{cm}^{-2}$ for the first device and $\mu^2 \approx 240,000\ \mathrm{cm}^{-2}\mathrm{V/s}$ and a charge impurity of $n^2_0 = 6 \cdot 10^8\ \mathrm{cm}^{-2}$ for the second device. We believe that the absence of fractional quantum Hall states at a field of B = 8 T is due to the relatively high density of charge impurities.

The vertical resistive line in Fig. S1h arises from the side gates, since the resistance is measured across the AD.

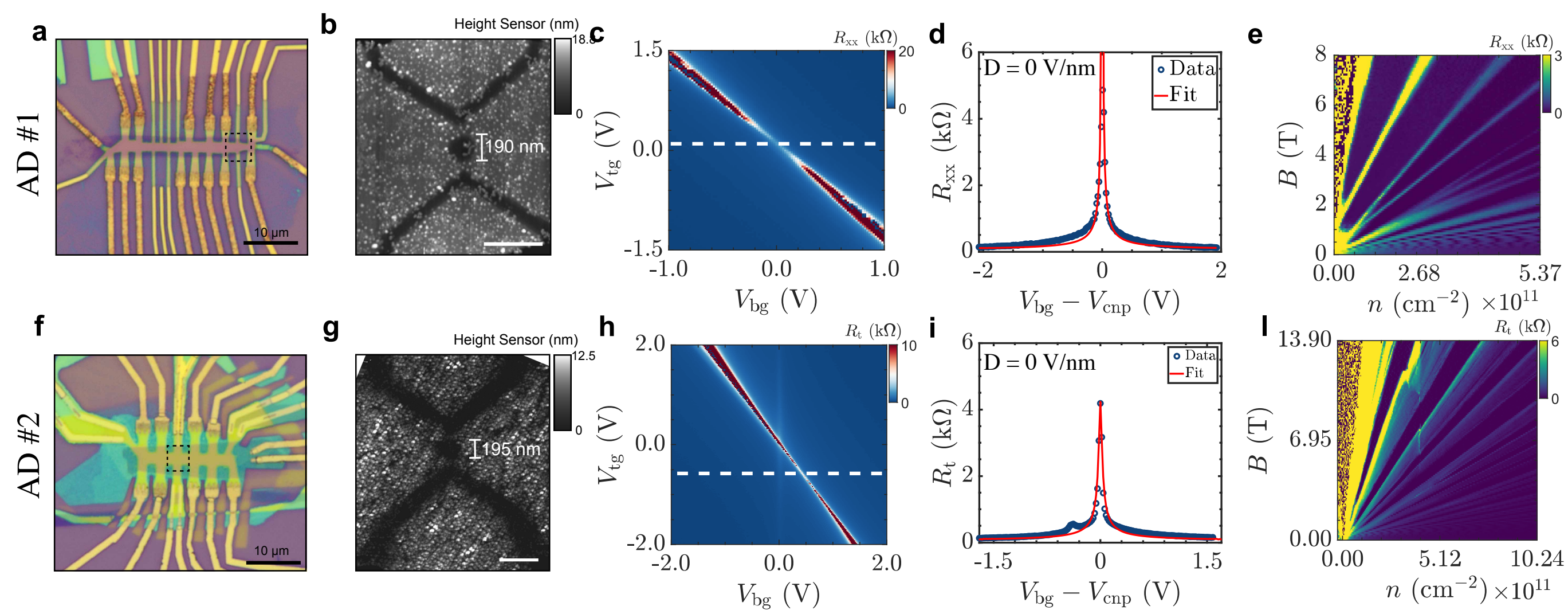

FIG. S1. **Devices characterization. a, f**, Optical microscope image of the devices AD #1 and AD #2, respectively. **b, g**, AFM height sensor image of the AD #1 and AD #2. The AD diameters are $D_{\mathrm{AD}\#1} = 190$ nm and $D_{\mathrm{AD}\#2} = 195$ nm. The scale bars are 400 nm. **c, g**, Longitudinal resistances measured outside the AD at $B = 0$ T as a function of the BG and TG voltage for AD #1, while for AD #2 it has been measured across the AD. **d, i**, Longitudinal resistance at zero displacement field for both devices as a function of the BG in blue and best fit in red. **e, l**, Longitudinal resistance and transmitted resistance fan diagram for AD #1 and AD #2 as a function of the carrier density n and the magnetic field B.

Figure S1e,l show the magneto-transport measurements for both devices. In BLG, Landau levels exhibit a fourfold degeneracy due to spin and valley degrees of freedom. Additionally, the two lowest orbital levels share the same

energy due to the LL energy dispersion, given by $E = \hbar\omega_c\sqrt{N(N-1)}$ where ω_c is the frequency of the cyclotron orbit. As the magnetic field increases, the Landau level degeneracy is lifted first for the spin, followed by valley splitting, and finally by the orbital number splitting [2–4].

In our setup, a voltage bias is applied to the device while the current is measured. We have some losses in the measured current because of the DC lines and low-pass filters, as illustrated in Fig. S2. Because of these filtering elements the resulting measured current is less than the one across the device. To account for this effect, we correct the current by applying a multiplicative factor when extracting the conductance. This factor is chosen such that the $\nu = 2$ quantum Hall plateau is properly quantized. In our case, the correction factor is $f = 1.02$.

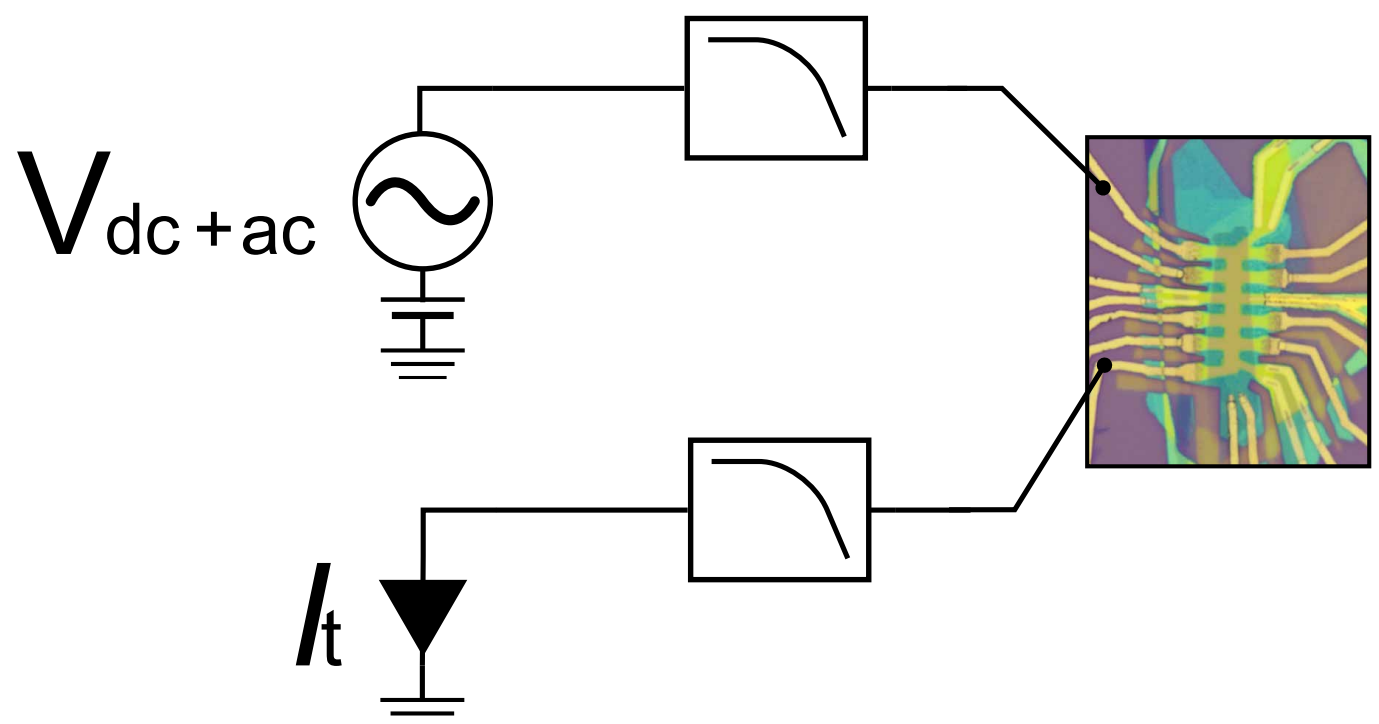

FIG. S2. **Current measurement.** Layout of the wiring for the conductance measurements.

B. Device Tuning

Silicon Gate Characterization

The graphene that extends outside the bottom graphite gate near the ohmic contacts is locally doped using the global silicon back gate. For each magnetic field B, the voltage applied to the silicon gate, V_{SiG}, is chosen by applying an ac voltage and monitoring the transmitted current I on a quantum Hall plateau, where all the current is transmitted. At specific V_{SiG} values, the transmitted currents saturates, as shown in Fig. S3a-b for the electron-side at $B = 13.5$ T and $V_{\mathrm{ac}} = 20\ \mu$V, and the hole-side at $B = 8$ T and $V_{\mathrm{ac}} = 3\ \mu$V, respectively. We fix the silicon gate V_{SiG} at the center of these plateau values. For the electron-side at $B = 13.5$ T $V_{\mathrm{SiG}} = 53.2$ V and for the hole-side at $B = 8$ T $V_{\mathrm{SiG}} = -46$ V.

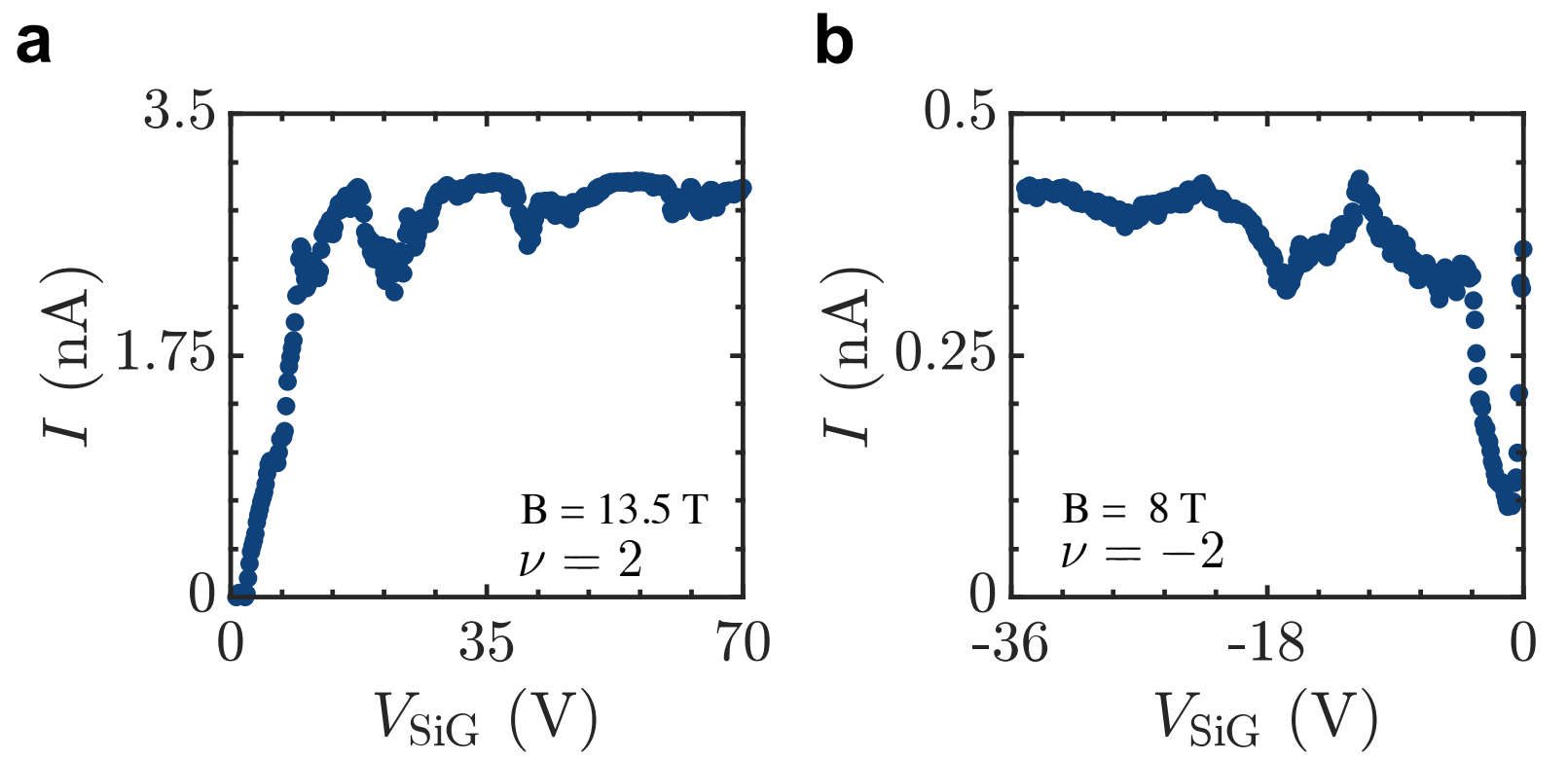

FIG. S3. **Silicon gate characterization.** Measured current I as a function of the silicon gate V_{SiG} for the electron-side at $B = 13.5$ T (**a**) and the hole-side at $B = 8$ T (**b**). For the electron-side at $B = 13.5$ T $V_{\mathrm{SiG}} = 53.2$ V and for the hole-side at $B = 8\mathrm{T}$ $V_{\mathrm{SiG}} = -46$ V. All the measurements have been performed in AD #2

Side Gates Characterization

The side gates enable control over the interaction between the extended edges and the AD–bound states. Applying a negative voltage to the side gates brings the edges closer together, thereby allowing tunneling.

A first characterization consists of fixing the BG voltage and measuring the transmitted resistance (R_{t}) and longitudinal resistance (R_{xx}) as functions of the top gate voltage V_{tg} and side gate voltage V_{sg}, as shown in Fig. S4a–b. For this measurement, the north side gate (NSG) and the south side gate (SSG) were swept symmetrically, $V_{\mathrm{SSG}} = V_{\mathrm{NSG}} = V_{\mathrm{sg}}$. The $\nu = 1$ plateau narrows as the side gate voltage decreases (Fig. S4a), while the plateau measured outside the AD remains constant (Fig. S4b). This confirms that the side gates are functioning properly.

However, due to fabrication imperfections, the side gates may not be perfectly symmetric with respect to the AD. At each filling factor, the side gates are tuned to identify the symmetric condition at which the downstream and upstream edges are equally coupled to the AD-bound states. To achieve this, the bulk filling factor is fixed at the edge of the plateau while the NSG and SSG are swept simultaneously. Examples are shown in Fig. S4c–d for $\nu = 1$ and Fig. S4e–f for $\nu = 5/3$.

For $\nu = 1$, we observe that both gates pinch almost symmetrically, similar to the behavior in quantum dot devices. In this case, the white dotted line indicates the voltages at which the side gates are symmetric. However, for $\nu = 5/3$, one gate exhibits a much stronger effect than the other. We attribute this asymmetry to localized impurities near one of the gates, which can locally trap electrons. We were not able to fully pinch the innermost edge because the required voltage would have been too high and could have risked damaging the device. In such cases, we use the value obtained at $\nu = 1$, assuming that, between filling factors, the coupling of both side gates to the edges does not change significantly. In both cases, we note that the longitudinal resistance R_{xx} outside the AD remains constant and equal to 0.

Finally, the voltage applied to the BG also controls the pinching of the side gates, as shown in S5, with a more negative bottom gate voltage that helps pinching the edges.

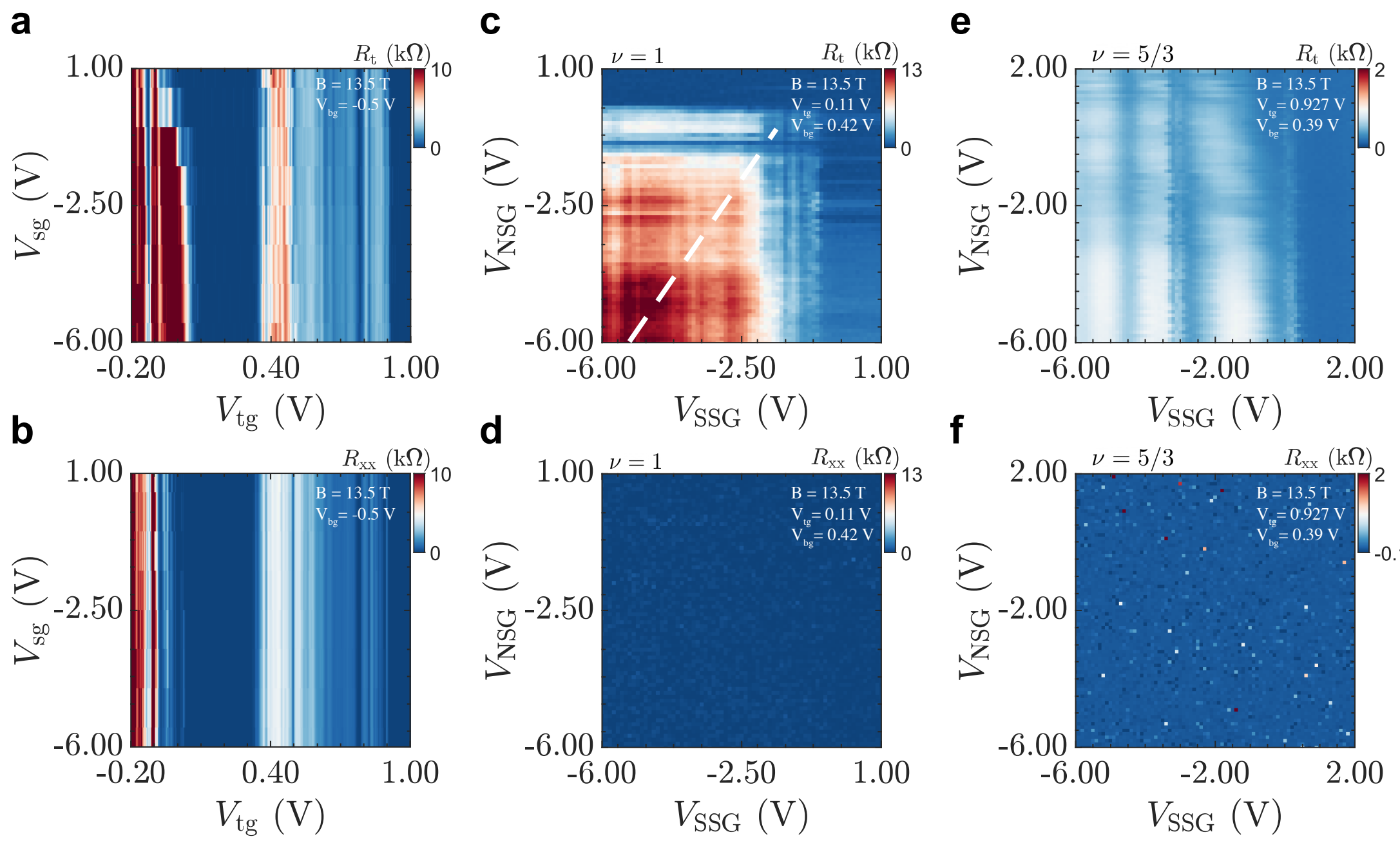

FIG. S4. **Side gates characterization a-b**, Transmitted (R_t) and longitudinal (R_{xx}) resistances in function of the top gate voltage V_{tg} and the side gate voltage V_{sg} at a BG of $V_{bg} = -0.5$ V. For this measurement the side gates were swept symmetrically, $V_{SSG} = V_{NSG} = V_{sg}$. **c-d**, Transmitted ($R_t$) and longitudinal ($R_{xx}$) resistances in function of the south side gate voltage V_{SSG} and the north side gate voltage V_{NSG} at $V_{bg} = 0.42$ V, $V_{tg} = 0.11$ V corresponding to the edge of the $\nu = 1$ plateau. The white dotted line in panel **c** indicates the voltages at which the side gates are symmetric. **e-f**, Transmitted (R_t) and longitudinal (R_{xx}) resistances in function of the south side gate voltage V_{SSG} and the north side gate voltage V_{NSG} at of $V_{bg} = 0.39$ V, $V_{tg} = 0.927$ V corresponding to the $\nu = 5/3$ plateau. All the measurements have performed with AD #2 and at $B = 13.5$ T.

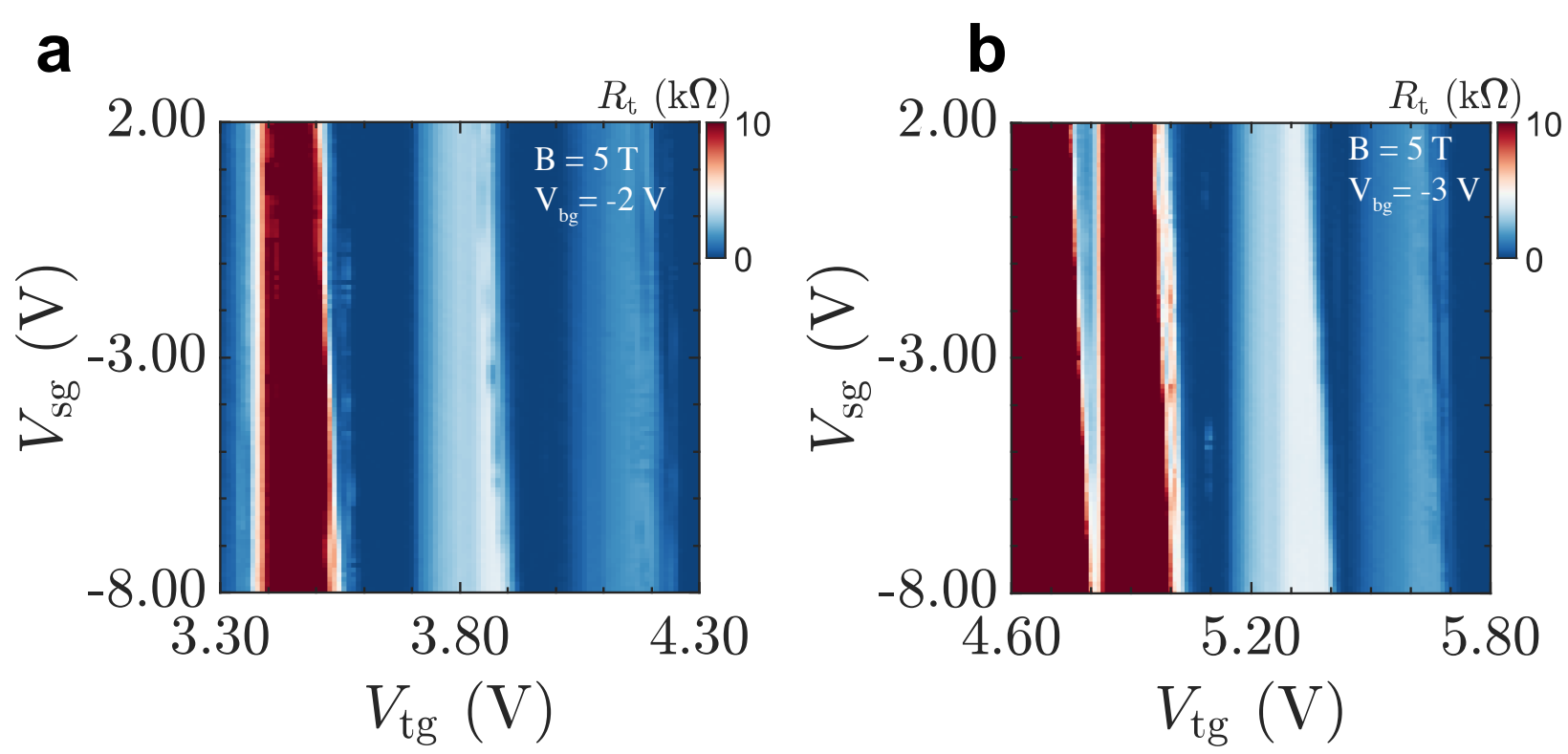

FIG. S5. **Side gates at different BG values a-b**, Transmitted resistance R_t in function of the top gate voltage V_{tg} and the side gate voltage V_{sg} at a BG of $V_{bg} = -2$V (**a**) and $V_{bg} = -3$V (**b**). The TG covers a range from $\nu = 1$ to $\nu = 4$. A more negative bottom gate voltage helps pinch the edges. The side gates were swept symmetrically, $V_{SSG} = V_{NSG} = V_{sg}$. All the measurements have been performed in AD #2.

C. Oscillations in the IQH regime

AD #1

Figure S6 shows the diagonal conductance G_d oscillations as a function of the TG voltage and magnetic field for filling factors $\nu = 1 - 4$ (a-d) and their 2D-FFT (e-h) for the first device AD #1.

All oscillations display Coulomb diamond behavior as shown in panels Fig. S6o-r. The white arrow in panel (o) indicates the direction of an increasing number of electrons bound to the AD. For each filling factor, we extract a charging energy, which is on the order of $300 - 600$ μeV and depends on the energy level spacing. It is highest at $\nu = 2$, followed by $\nu = 4$, $\nu = 1$, and finally $\nu = 3$. This trend aligns with the Landau level structure in bilayer graphene, where the Landau level gaps at $\nu = 4$ and $\nu = 2$ are the largest [5]. To estimate the energy level spacing without considering electron-electron interactions, we can use the expression $\delta\epsilon = 2\hbar v/D_{AD}$ with $\hbar$ is the reduced Planck's constant and $v = -\frac{1}{eB}\frac{\partial V}{\partial r}$ is the drift velocity of the charge carriers on the AD potential V. In graphene interferometers, a typical value for the drift velocity is $v = 10^5$ m/s [1, 6–9]. Assuming an AD diameter D_{AD} of 300 nm, the energy level spacing is $\delta\epsilon \approx 450$ μeV. This estimated spacing aligns well with experimental observations without the need for electron-electron interactions. Moreover, for $\nu = 2$, we observe that the level spacing energy increases as the TG voltage increases. This behavior is consistent with a modification of the AD potential slope: increasing the slope increases the drift velocity of the charge carriers and therefore leads to a larger level spacing.

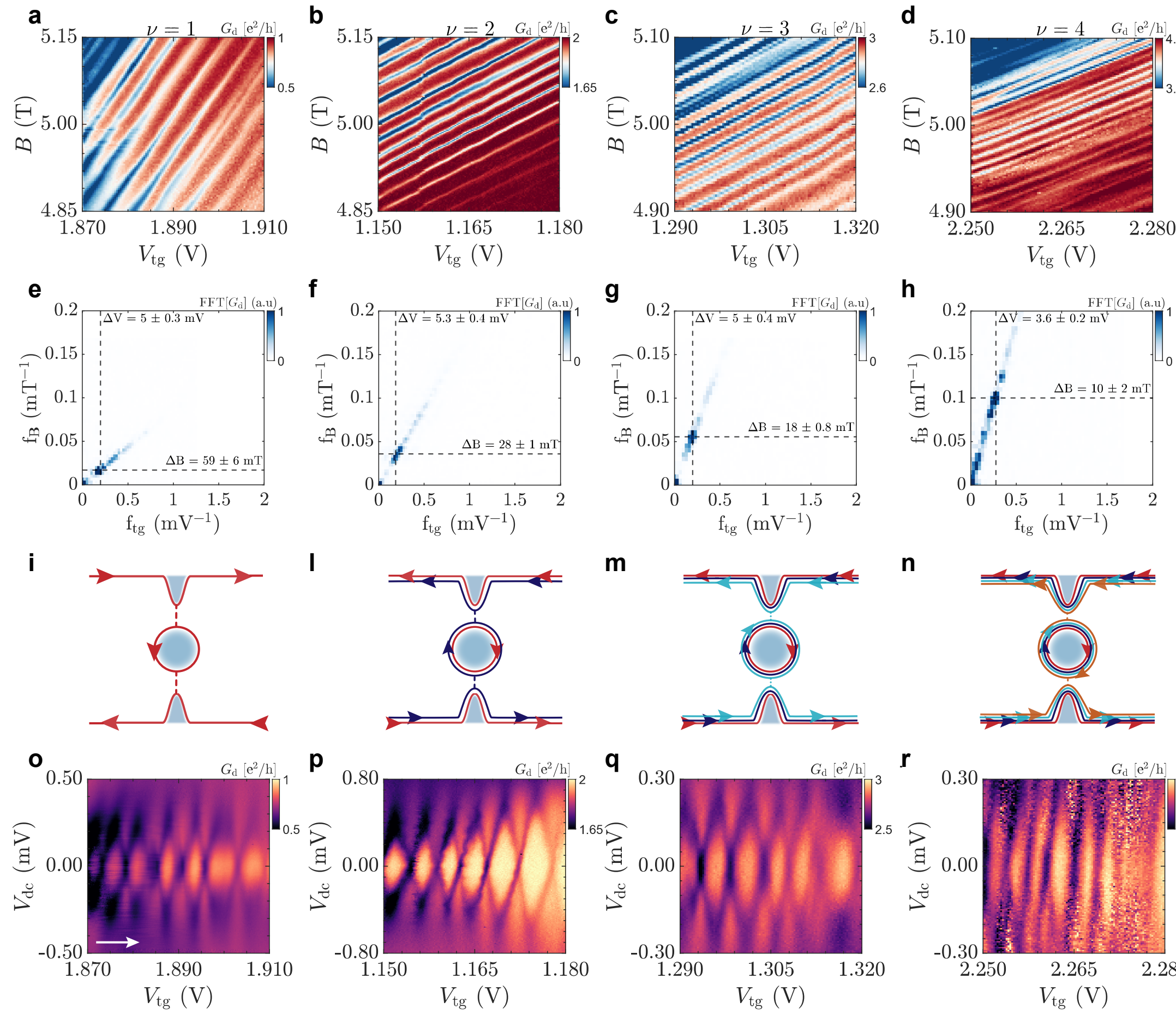

FIG. S6. **IQH oscillations for AD #1.** **a-d**, Diagonal conductance G_{d} oscillations as a function of the TG voltage and magnetic field for filling factors $\nu = 1 - 4$. **e-h**, 2D-FFT of the oscillations. **g-n** Schematic of the tunneling edge. **o-r**, Diagonal conductance G_{d} oscillations as a function of TG voltage and DC bias, displaying the characteristic Coulomb diamonds. The white arrow in panel **o** indicates the AD-bound electrons increasing direction.

AD #2

Figure S7 shows the diagonal conductance G_d oscillations as a function of the TG voltage and magnetic field for filling factors $\nu = 1, 2$ and 4 (a–c) and their corresponding 2D-FFT spectra (d–f), measured for the second device (AD #2) at $B = 13.5$ T. From the Source-Drain measurements (g-i) we can extract the charging energy. It can be observed that the charging energies are much smaller at $B = 13.5$ T compared to lower magnetic fields (Fig. S8), as expected from the scaling $\Delta\epsilon \propto 1/B$. Furthermore, for $\nu = 1$ and $\nu = 4$, it appears that the device may not be operating in the single-level hopping regime ($k_BT \ll \Delta\epsilon$), as Coulomb diamonds are difficult to resolve. In these cases, transport is likely dominated by multilevel hopping ($k_BT \geq \Delta\epsilon$). This can also explain the observed quasiperiodic oscillations.

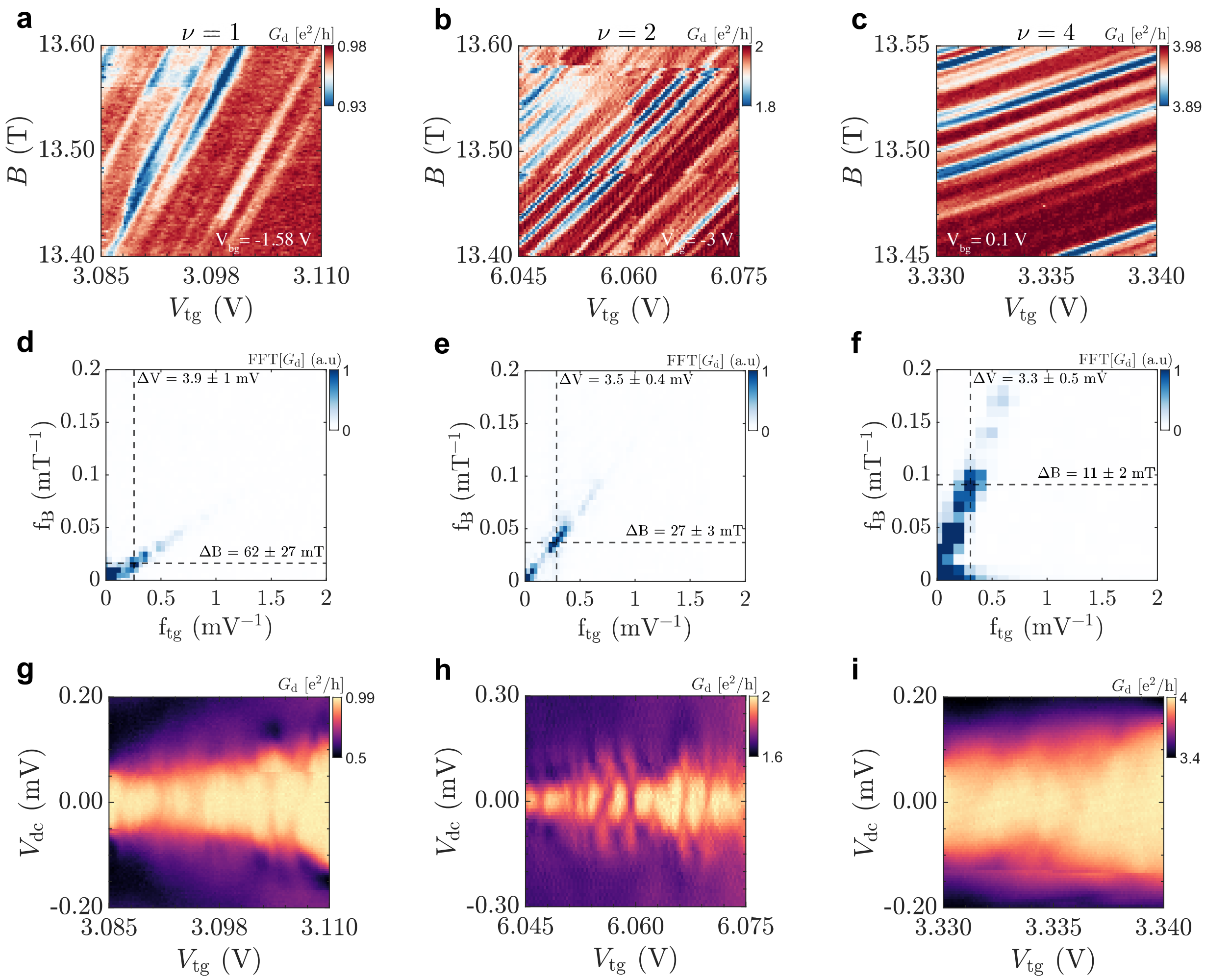

FIG. S7. **IQH oscillations for AD #2. a-c**, Diagonal conductance G_d oscillations as a function of the BG voltage and magnetic field for filling factors $\nu = 1, 2$ and 4. **d-f**, 2D-FFT of the oscillations. **g-i**, Diagonal conductance G_d oscillations as a function of TG voltage and DC bias.

Figure S8 shows the diagonal conductance G_d oscillations as a function of the TG voltage and magnetic field for filling factors $\nu = -2, 1, 2,$ and 4 (a–d), together with their corresponding 2D-FFT spectra (e–h), measured for the second device (AD #2). On the hole side, at $\nu = -2$, the oscillations display an opposite slope, as expected from density oscillations. In this case, we changed the contact configuration to account for the opposite chirality of the edge states.

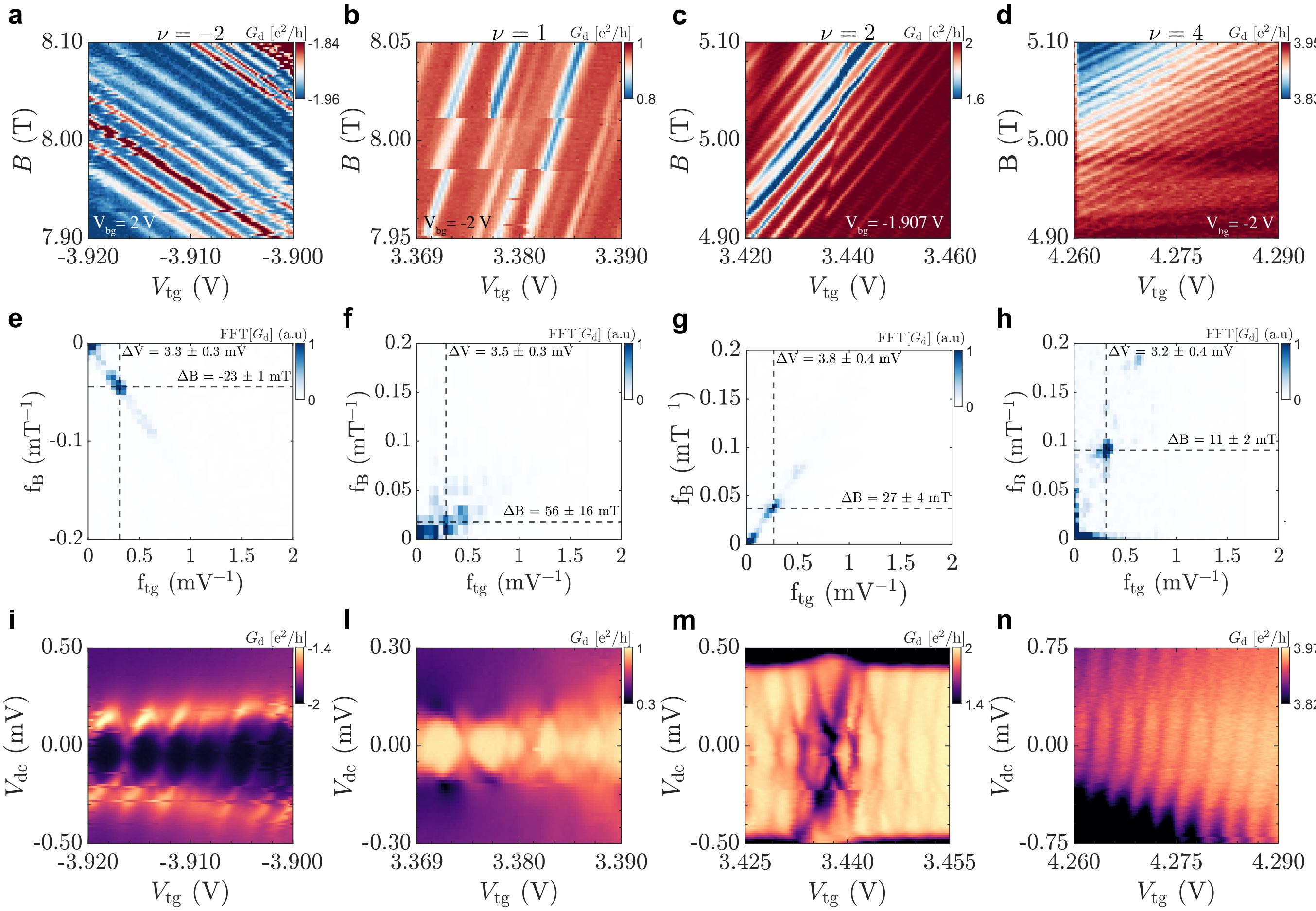

FIG. S8. **Lower field IQH oscillations for AD #2. a-d**, Diagonal conductance G_d oscillations as a function of the TG voltage and magnetic field for filling factors $\nu = -2, 1, 2$ and 4. **e-h**, 2D-FFT of the oscillations, the insert schematically shows the interfering edge. **i-n**, Diagonal conductance G_d oscillations as a function of TG voltage and DC bias. The measurements for $\nu = 4$ at $B = 5$ T have been taken at a temperature of T = 300 mK.

D. Oscillation in Bottom Gate

As mentioned in the main text, another way to achieve oscillations is by varying the BG while keeping the TG constant. However, the drawback of this method is that the AD potential and the side gates trenches are primarily controlled by the BG. Consequently, a small variation in the BG has a much larger effect on the potential landscape compared to a similar variation in the TG.

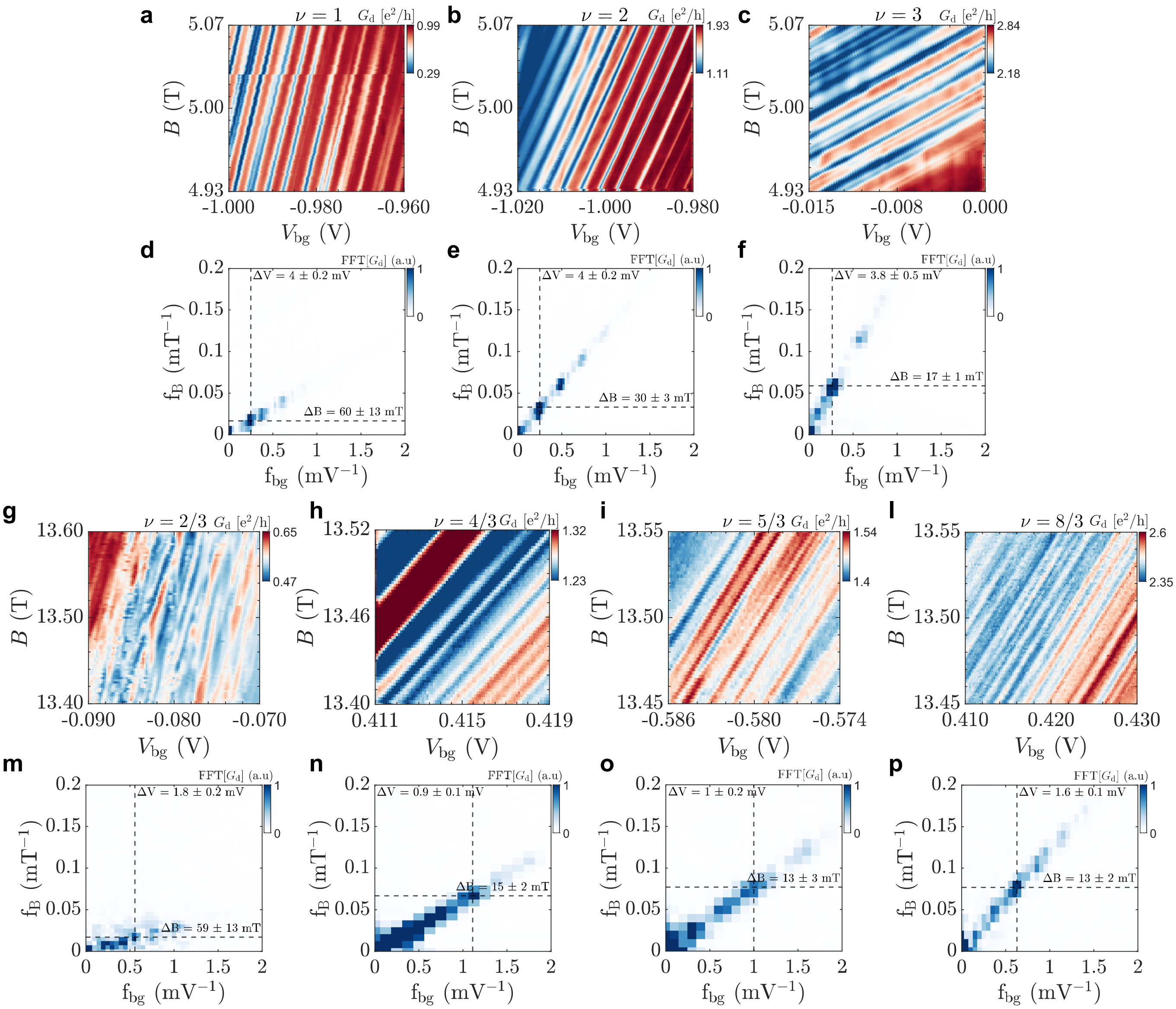

FIG. S9. **Oscillations in bottom gate. a-c**, Diagonal conductance G_d oscillations as a function of the bottom gate voltage and magnetic field for filling factors $\nu = 1 - 3$ for the first device (AD #1). **d-f**, 2D-FFT of the oscillations. **g-l**, Diagonal conductance G_d oscillations as a function of the bottom gate voltage and magnetic field for filling factors $\nu = 2/3, 4/3, 5/3$ and 8/3 for the second device (AD #2). **d-f**, 2D-FFT of the oscillations.

Figure S9a-c shows oscillations obtained in the BG at $\nu = 1 - 3$ for the first device (AD #1), with similar results to those observed in the TG. As shown in Fig. S9d-f, the magnetic field period closely matches the one obtained for TG oscillations, as reported in the main text and in Fig. S6. However, the BG period differs due to the thinner bottom hBN layer, which results in a larger capacitance. Figure S9g-l shows the same measurements performed on AD #1 for fractional filling factors, $\nu = 2/3, 4/3, 5/3$ and 8/3, with their respective 2D-FFT, Fig. S9g-l.

Figure S10 reports the periods and the charge quasiparticle charge as a function of filling factor. As for the observations for TG oscillations, we find that the extracted charge follows the same values as the TG. Figure S10c-d shows the ratio of the bottom-gate period to the magnetic-field period, normalized by the bottom-gate capacitance per unit area and ϕ_0/e, as a function of the filling factor ν with its relative error, respectively. The dotted orange lines are guides to the eye. As for the TG, the oscillations exhibit a slope corresponding to the filling factor ν.

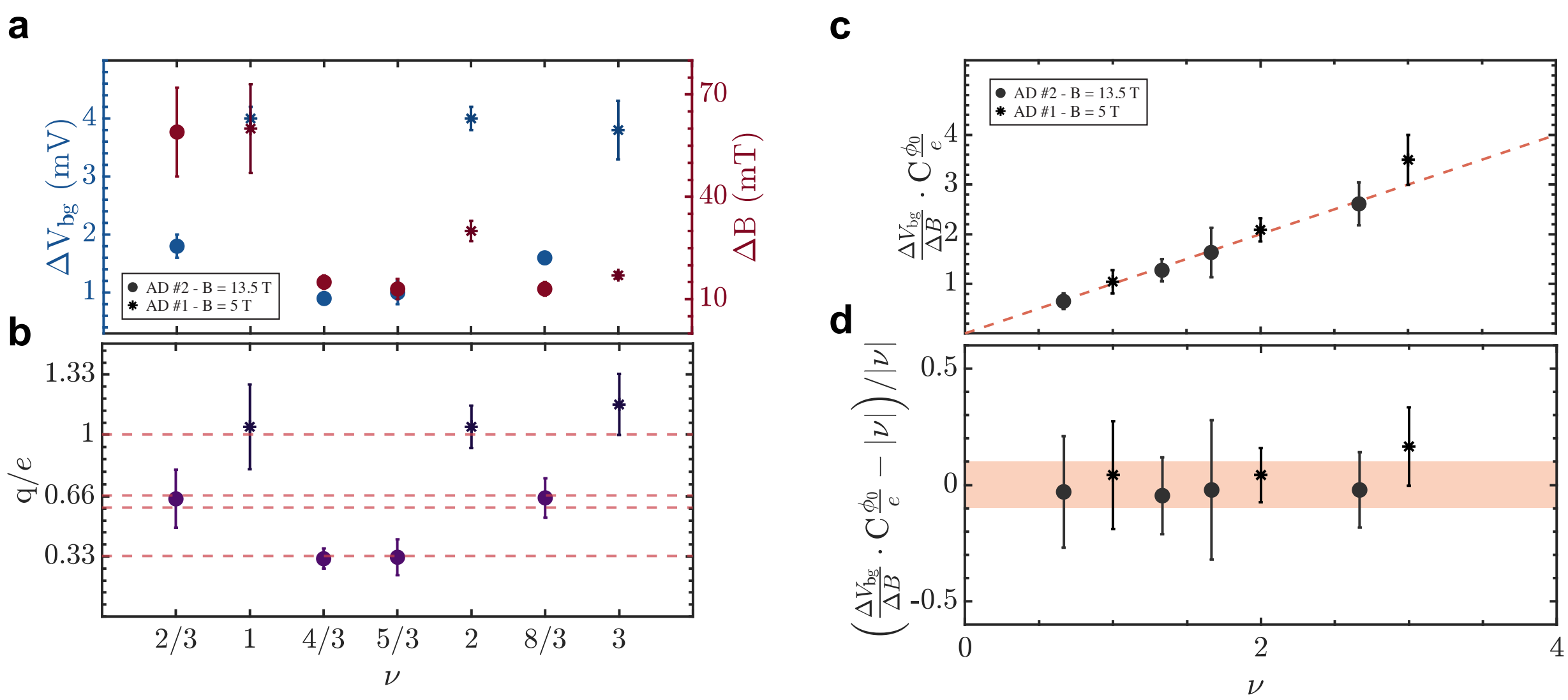

FIG. S10. **Charge measurement using the bottom gate.** **a** Bottom-gate period ΔV_{bg} (blue) and magnetic-field period ΔB (red) as a function of filling factor ν for oscillations measured at different magnetic fields and on two devices. **b**, Quasiparticle charge as a function of filling factor, computed using Eq. **??**. **c**, Ratio of the bottom-gate period to the magnetic-field period, normalized by the bottom-gate capacitance per unit area and ϕ_0/e, as a function of the filling factor ν. The dotted orange lines are guides to the eye. The oscillations exhibit a slope corresponding to the filling factor ν. **d**, Relative error of the ratio of the bottom-gate period to the magnetic-field period minus the filling factor. The shaded orange band marks the $\pm 10\%$ interval.

E. Oscillation in Area

Another way to induce gate oscillations is by modifying the AD potential, which in turn changes the AD area. This can be achieved by sweeping both the TG and the BG simultaneously while maintaining a constant bulk filling factor. In fact, the AD potential is mainly controlled by the BG; if the BG voltage decreases (TG voltage increases to keep a constant bulk filling factor), the AD potential will increase.

As the AD potential increases, so does its area, and to preserve a constant flux, the bound edge states shift to higher energy. An oscillation occurs when the chemical potential of an AD-bound state aligns with the source chemical potential, as schematically illustrated in S11a.

The equation for constant density lines is given by

$$V_{\mathrm{tg}} = \alpha V_{\mathrm{bg}} + \beta \tag{2}$$

where $\beta = \frac{e^2 \nu B}{h C_{\mathrm{tg}}}$ defines the filling factor, and C_{tg} is the TG capacitance per unit area. The value α is the ratio of the BG and TG capacitance; for AD #1 we have used $\alpha = -1.34$ and for AD #2 $\alpha = -1.465$.

Figure S11b-c illustrates the area oscillations for $\nu = 2$ in AD #1 (b) and for $\nu = 5/3$ in AD #2. The oscillations exhibit an opposite slope compared to density oscillations due to the opposite direction in which the energy levels move with respect to the increasing direction of the area, as indicated by the white arrow in Fig. S11b

The 2D-FFT analysis in Fig. S11d-e reveals a magnetic field period comparable to that observed in density oscillations, while the gate voltage period appears larger with a periodicity of $\Delta V \approx 39$ mV, which is almost nine times larger than the one obtained when changing the density. This suggests that the energy levels of the AD-bound states shift up by at least one level every nine electron jumps, which is consistent with a 5% overestimation of the electron charge using only the TG voltage. To confirm this theory, we performed oscillations using only BG (section D) that show an estimate of the tunneling charge of about 5% less than the electron charge.

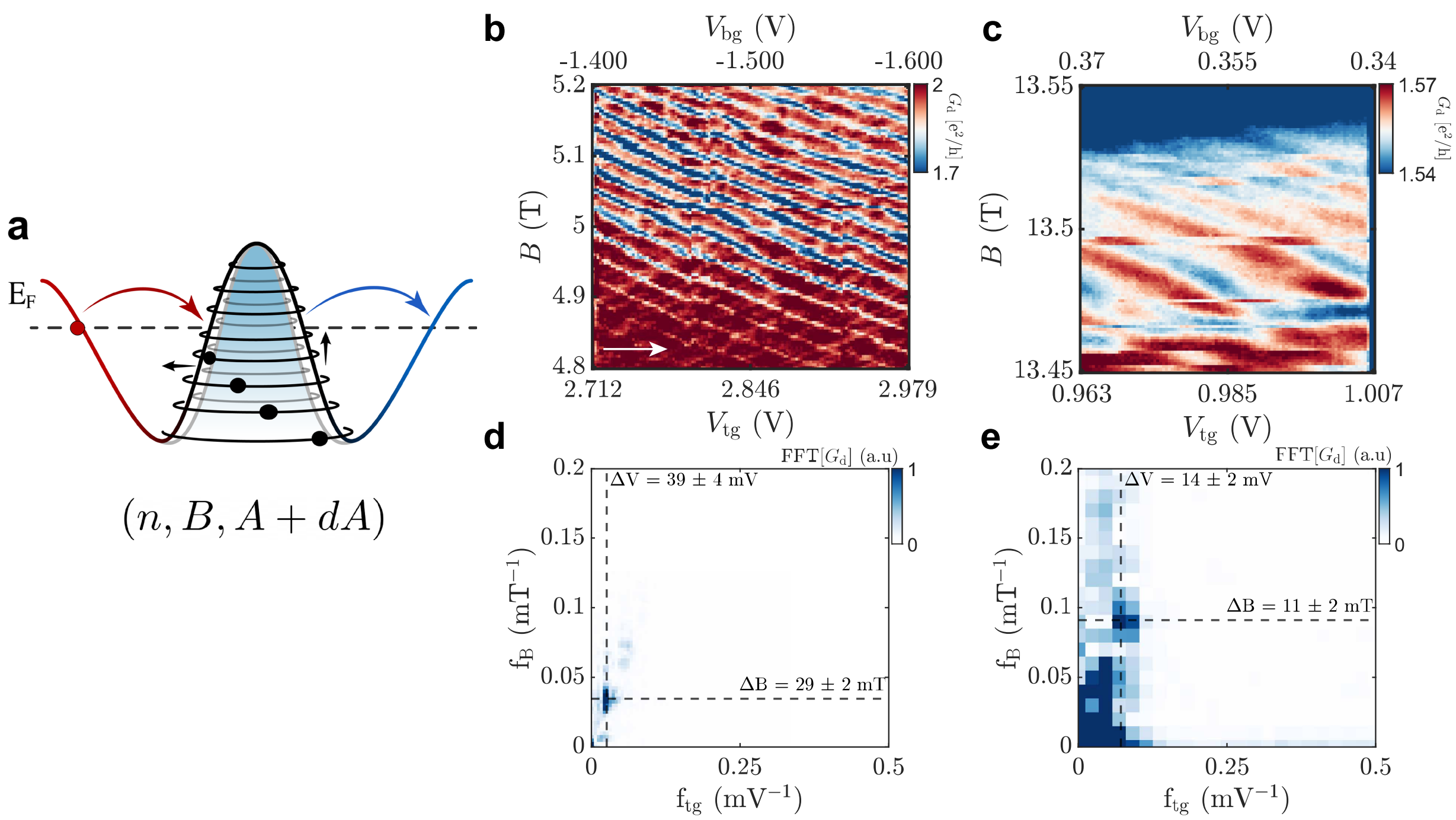

FIG. S11. **Oscillations in the AD area.** **a** Schematic of the tunneling when changing the AD area. Diagonal conductance G_{d} oscillations as a function of the area and magnetic field for filling factor $\nu = 2$ in AD #1 (**b**) and for $\nu = 5/3$ in AD #2 (**c**). **d-e**, 2D-FFT of the oscillations. The white arrow in panel **b** indicates the area increasing direction.

F. Outer Edge at $\nu = 2$

As the constriction filling factor continues to decrease, the inner edge can become completely reflected, allowing the outer edge to start interfering. In this case, we expect the magnetic field oscillation period to be $\phi_0/\nu_{\rm int}$ with $\nu_{\rm int} = \nu_{\rm b} - 1$.

Figure S12a shows oscillations for the outer edge at a filling factor of 2 ($\nu_{\rm b} = 2$, $\nu_{\rm int} = 1$). From the 2D-FFT, we extract a magnetic field period of $\Delta B = 73 \pm 10$ mT, which is similar to that of the inner edge at filling factor 1. This corresponds to a flux period of ϕ_0 and an AD diameter of $D = 269 \pm 18$ nm. Since the oscillations are gate-induced, we cannot directly extract the interfering charge.

Source-drain measurements, shown in Fig. S12c, still display a Coulomb-dominated pattern with a charge excitation energy of $E = 210 \pm 10$ μeV.

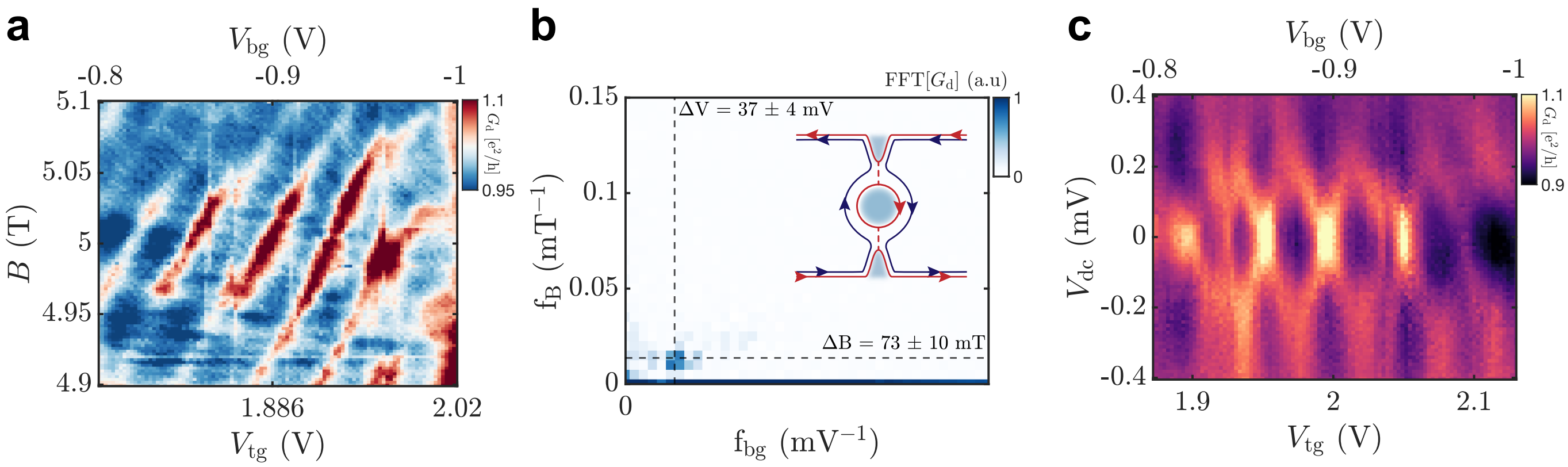

FIG. S12. **Oscillations in the outer edge of $\nu = 2$** **a** Diagonal conductance $G_{\rm d}$ oscillations as a function of the AD area magnetic field for the outer edge of filling factor $\nu = 2$. **b**, 2D-FFT of the oscillations. The insert schematically shows the outer edge interfering while the inner one is fully reflected. **c**, Diagonal conductance $G_{\rm d}$ oscillations as a function of the AD area DC bias.

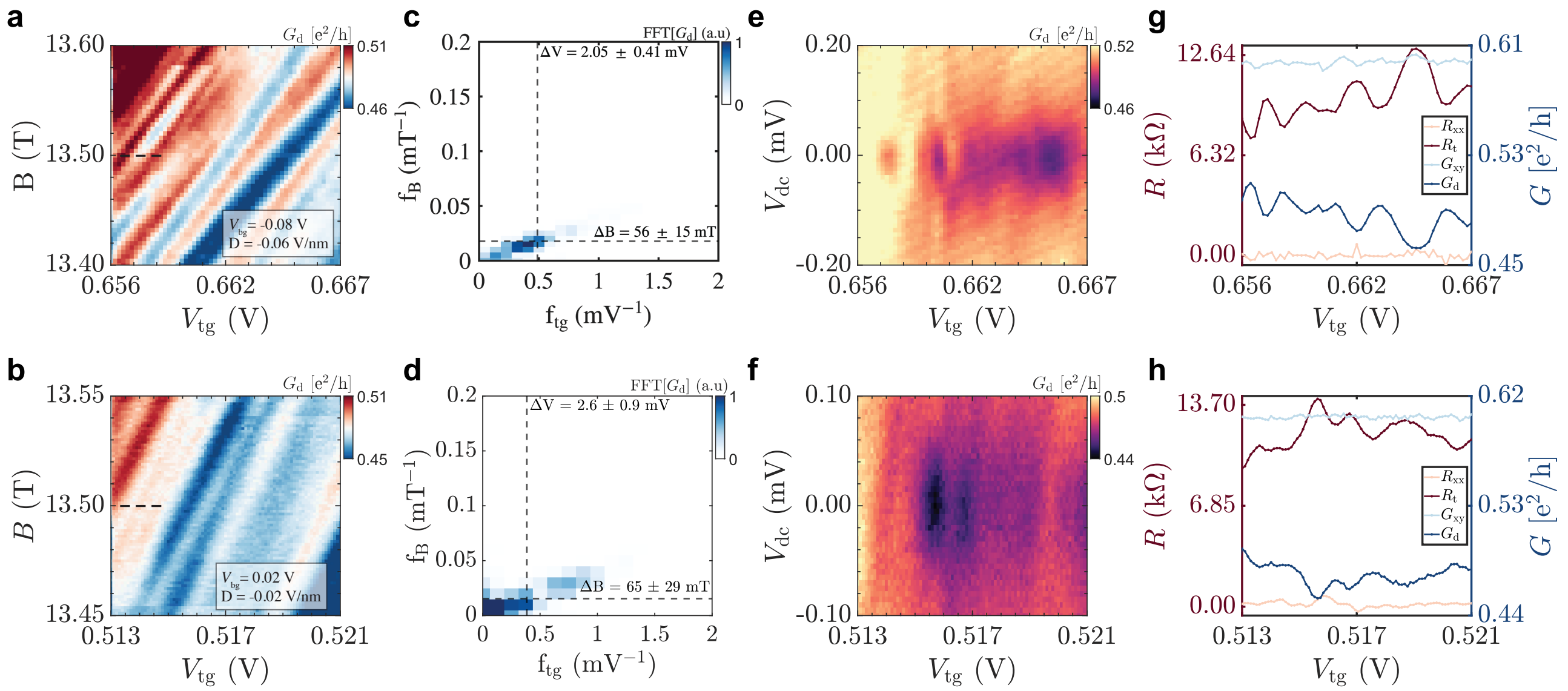

FIG. S13. **Oscillations at $\nu = 3/5$. a-b**, Diagonal conductance $G_{\rm d}$ oscillations as a function of the TG voltage and magnetic field for filling factor $\nu = 3/5$ at different displacement fields D. **c-d**, 2D-FFT of the oscillations. **e-f**, Diagonal conductance $G_{\rm d}$ oscillations as a function of TG voltage and DC bias. **g-h**, Transmitted resistance measured across the AD ($R_{\rm t}$ in dark red), longitudinal resistance measured outside the AD ($R_{\rm xx}$ in light red), diagonal conductance ($G_{\rm d}$ in dark blue) and Hall conductance ($G_{\rm xy}$ in light blue) as a function of TG voltage. The oscillations are taken along the dotted lines in their respective panels **a-b**.

G. Oscillations in the FQH regime

In this section, we report oscillations measured at different fractional filling factors. Each oscillation is identified by the bottom-gate voltage $V_{\rm bg}$ at which it was acquired, together with the corresponding displacement field, D. Almost all oscillations occur at a low displacement field where the orbital number remains the same. For $\nu = 5/3$, oscillations were also taken at larger displacement fields without any detectable variation, suggesting that the displacement field primarily modifies the potential slope of the AD without altering the nature of the oscillations.

For each set of oscillations, we have also reported the source–drain bias dependence. The latter provides information on the level spacing of the underlying states. From each fractional filling factor, we extract a charging energy on the order of 50 μeV, which is nearly an order of magnitude smaller than that obtained for integer oscillations. This behavior is expected, since fractional states generally exhibit smaller energy gaps. Moreover, the charging energy (or level spacing) scales inversely with both the magnetic field and the quasiparticle charge, $\delta\epsilon \propto -\frac{1}{e^* B}$. As a result, at higher magnetic fields, the energy spacing becomes smaller. At the same time, the reduced quasiparticle charge e^* in fractional states increases the spacing, making fractional oscillations comparatively easier to observe. However, as shown in Fabry–Pérot interferometer and STM experiments [7, 10], the bias dependence is not a unique fingerprint of the oscillation mechanism. In some cases, conventional Coulomb diamonds are observed, consistent with the IQH regime where the AD is filled and tunneling is suppressed. In other cases, inverted diamonds appear, characterized by a conductance minimum at zero bias. Figure S15l shows the source–drain bias dependence of the oscillation at $\nu = 4/3$, also discussed in the main text. It can be observed that the zero-bias conductance oscillation exhibits a transition from a peak to a dip. This behavior suggests a phase shift in the oscillation pattern, which is consistent with an Aharonov–Bohm–like mechanism rather than a purely Coulomb-dominated regime. However, due to the limited extent of the fractional plateaus, systematic exploration across the plateau was not possible, preventing a definitive conclusion.

Finally, for each oscillation, we also present a line cut as a function of the top-gate voltage. The transmitted resistance across the AD ($R_{\rm t}$, dark red), the longitudinal resistance outside the AD ($R_{\rm xx}$, light red), the diagonal conductance ($G_{\rm d}$, dark blue), and the Hall conductance ($G_{\rm xy} = R_0/(R_{\rm d} - R_{\rm t})$, light blue, with R_0 the quantum of resistance) are simultaneously displayed. Oscillations are consistently measured at the center of the fractional plateau, where $R_{\rm xx} = 0$ and $G_{\rm xy}$ is constant. In this regime, the oscillations in $R_{\rm xx}$ and $G_{\rm d}$ are fully correlated,

confirming that they originate from the AD.

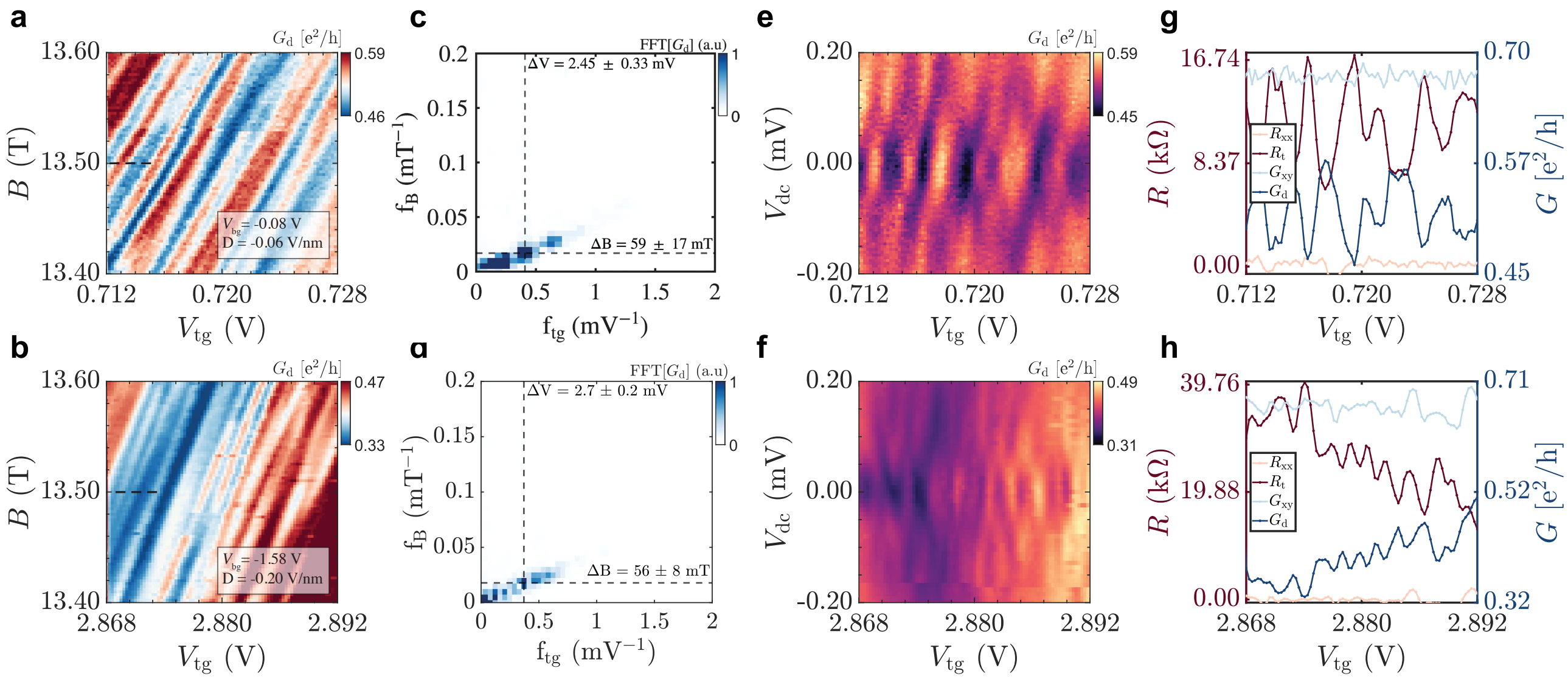

FIG. S14. **Oscillations at $\nu = 2/3$. a-b**, Diagonal conductance G_d oscillations as a function of the TG voltage and magnetic field for filling factor $\nu = 2/3$ at different displacement fields D. **c-d**, 2D-FFT of the oscillations. **e-f**, Diagonal conductance G_d oscillations as a function of TG voltage and DC bias. **g-h**, Transmitted resistance measured across the AD (R_t in dark red), longitudinal resistance measured outside the AD (R_xx in light red), diagonal conductance (G_d in dark blue) and Hall conductance (G_xy in light blue) as a function of TG voltage. The oscillations are taken along the dotted lines in their respective panels **a-c**.

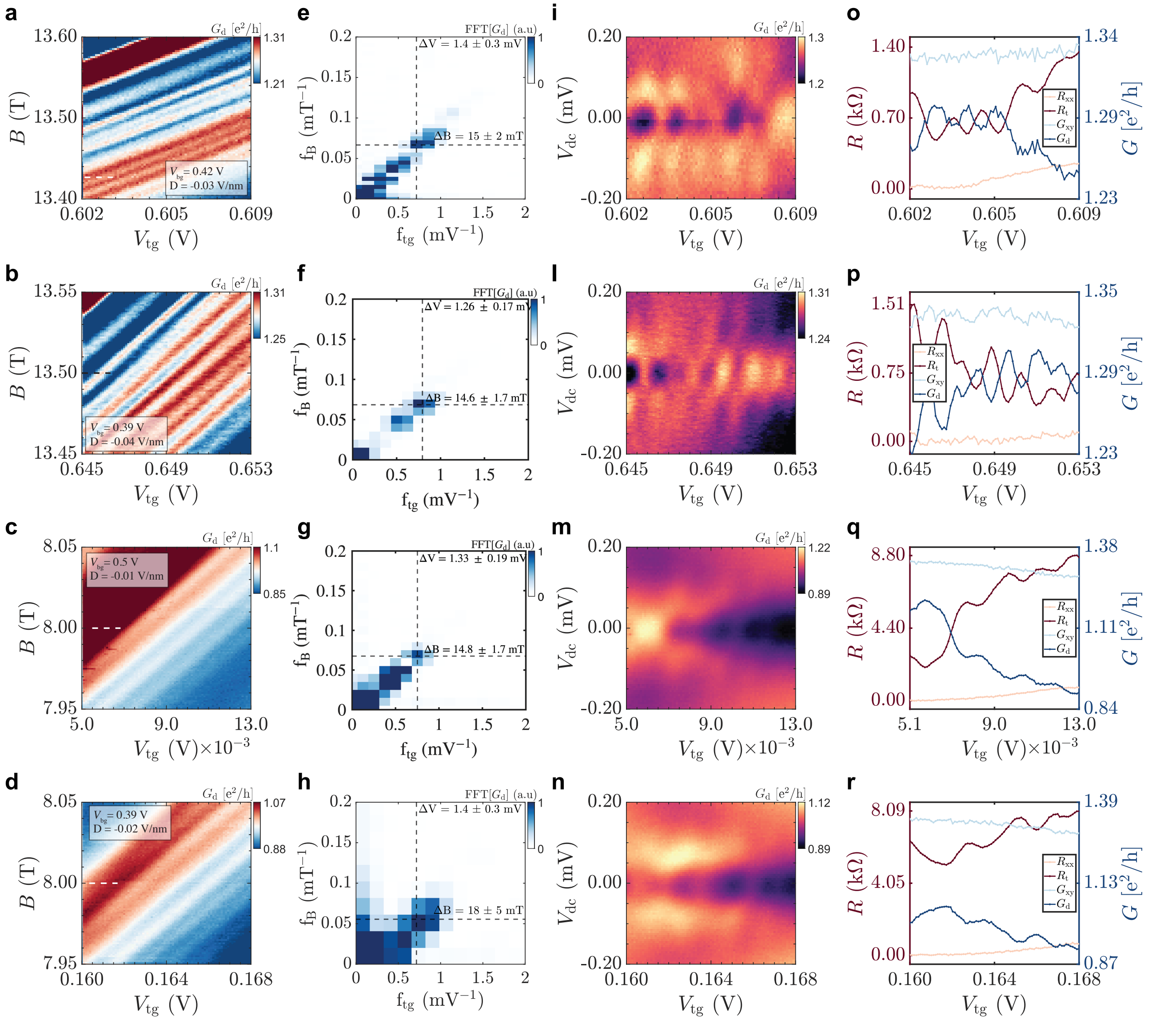

FIG. S15. **Oscillations at $\nu = 4/3$. a-d**, Diagonal conductance G_d oscillations as a function of the TG voltage and magnetic field for filling factor $\nu = 4/3$ at different displacement fields D and at different magnetic fields. **e-h**, 2D-FFT of the oscillations. **i-n**, Diagonal conductance G_d oscillations as a function of TG voltage and DC bias. **o-r**, Transmitted resistance measured across the AD (R_t in dark red), longitudinal resistance measured outside the AD (R_xx in light red), diagonal conductance (G_d in dark blue) and Hall conductance (G_xy in light blue) as a function of TG voltage. The oscillations are taken along the dotted lines in their respective panels **a-d**.

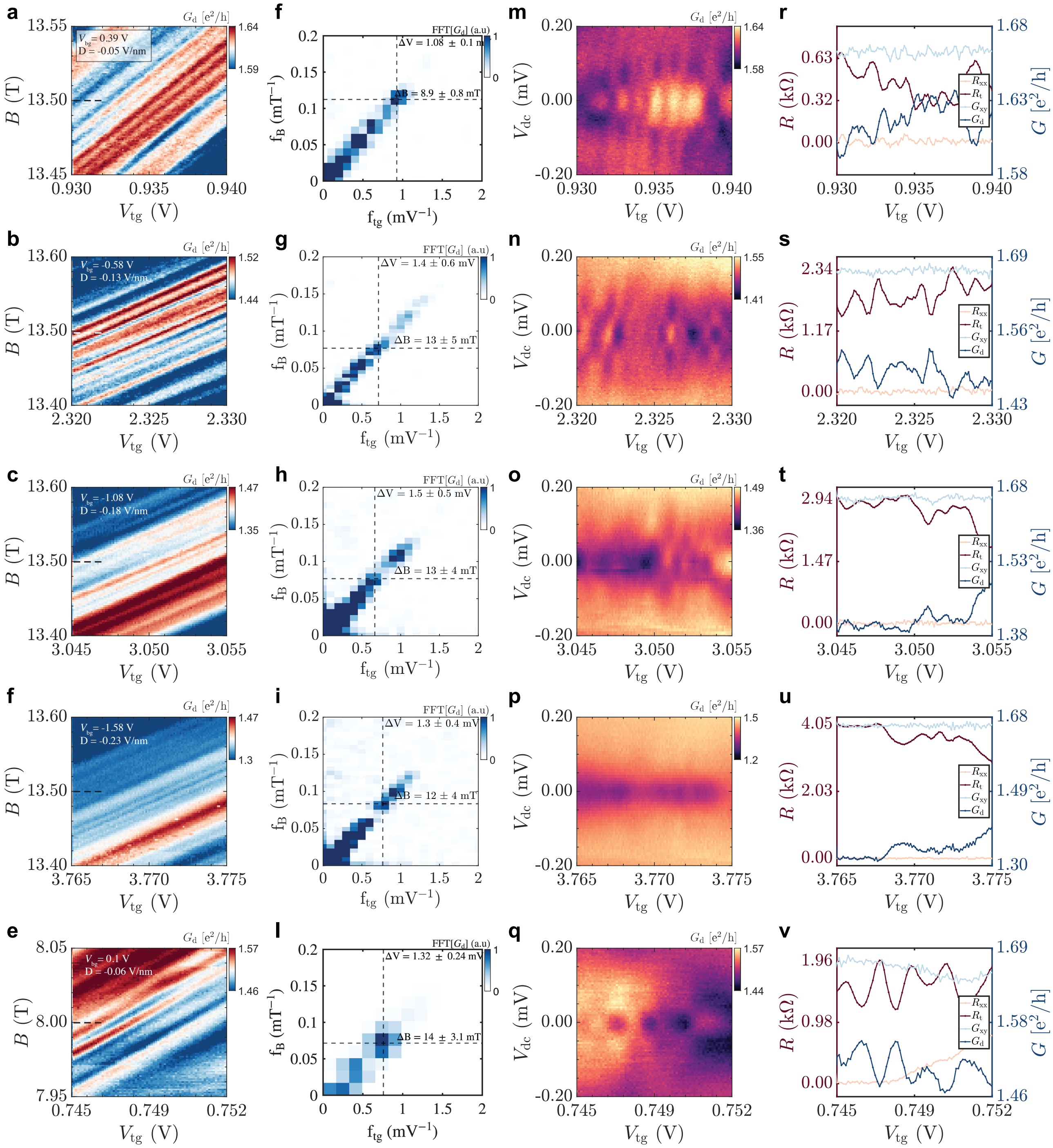

FIG. S16. **Oscillations at $\nu = 5/3$. a-e**, Diagonal conductance G_d oscillations as a function of the TG voltage and magnetic field for filling factor $\nu = 5/3$ at different displacement fields D and at different magnetic fields. **f-i**, 2D-FFT of the oscillations. **l-q**, Diagonal conductance G_d oscillations as a function of TG voltage and DC bias. **r-v**, Transmitted resistance measured across the AD (R_t in dark red), longitudinal resistance measured outside the AD (R_{xx} in light red), diagonal conductance (G_d in dark blue) and Hall conductance (G_{xy} in light blue) as a function of TG voltage. The oscillations are taken along the dotted lines in their respective panels **a-e**.

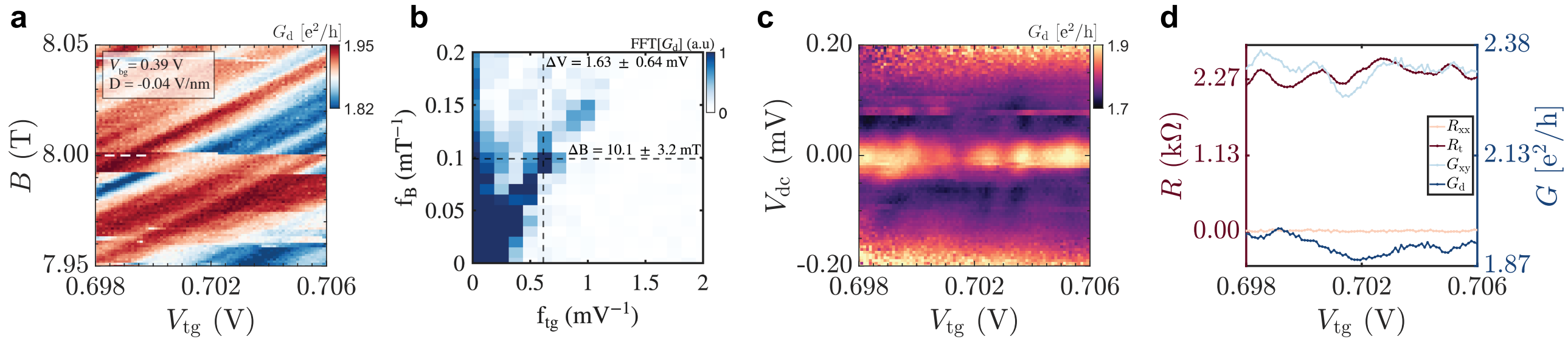

FIG. S17. **Oscillations at $\nu = 7/3$.** **a**, Diagonal conductance G_d oscillations as a function of the TG voltage and magnetic field for filling factor $\nu = 7/3$ at different displacement fields D. **b**, 2D-FFT of the oscillations. **c**, Diagonal conductance G_d oscillations as a function of TG voltage and DC bias. **d**, Transmitted resistance measured across the AD (R_t in dark red), longitudinal resistance measured outside the AD (R_xx in light red), diagonal conductance (G_d in dark blue) and Hall conductance (G_xy in light blue) as a function of TG voltage. The oscillations are taken along the dotted lines in the respective panel **a**.

Oscillations at $\nu = 8/3$

Of particular importance are the oscillations at filling factor $\nu = 8/3$, as they display two distinct types of periodicity. As highlighted in Fig. S18a, the dominant oscillation corresponds to a quasiparticle tunneling charge of $q/e = 2/3$ and a flux periodicity of $\Delta\phi = \phi_0/4$, while in certain regions oscillations with smaller periodicity can be observed, corresponding instead to a quasiparticle tunneling charge of $q/e = 1/3$ and a flux periodicity of $\Delta\phi = \phi_0/8$. The 2D-FFT reveals a main spectral peak corresponding to the longer-period ($2e/3$) oscillation, alongside a weaker secondary peak associated with the $e/3$ oscillations. To further resolve this secondary peak, we perform a 2D-FFT over a restricted region, indicated by the dotted white line in Fig. S18**a**. The peak is clearly distinguishable and corresponds to a periodicity of $\phi_0/8$ with a charge $e/3$. We interpret this as evidence that at $\nu = 8/3$ the fundamental quasiparticles carry charge $e/3$, and that this charge should, in principle, be observable. However, as discussed in the main text, the oscillation amplitude may depend on whether an even or odd number of quasiparticles is added to the AD, with one process dominating over the other. This mechanism can lead to the effective observation of a doubled charge, so that in most regimes only the stronger $2e/3$ oscillation is detected.

Finally, we note that the expected magnetic field oscillation period of the $e/3$ state, $\Delta B \approx 7$ mT, is close to the minimum resolution achievable with our present magnet power supply. This instrumental limitation makes the direct detection of the smallest-period oscillation particularly challenging.

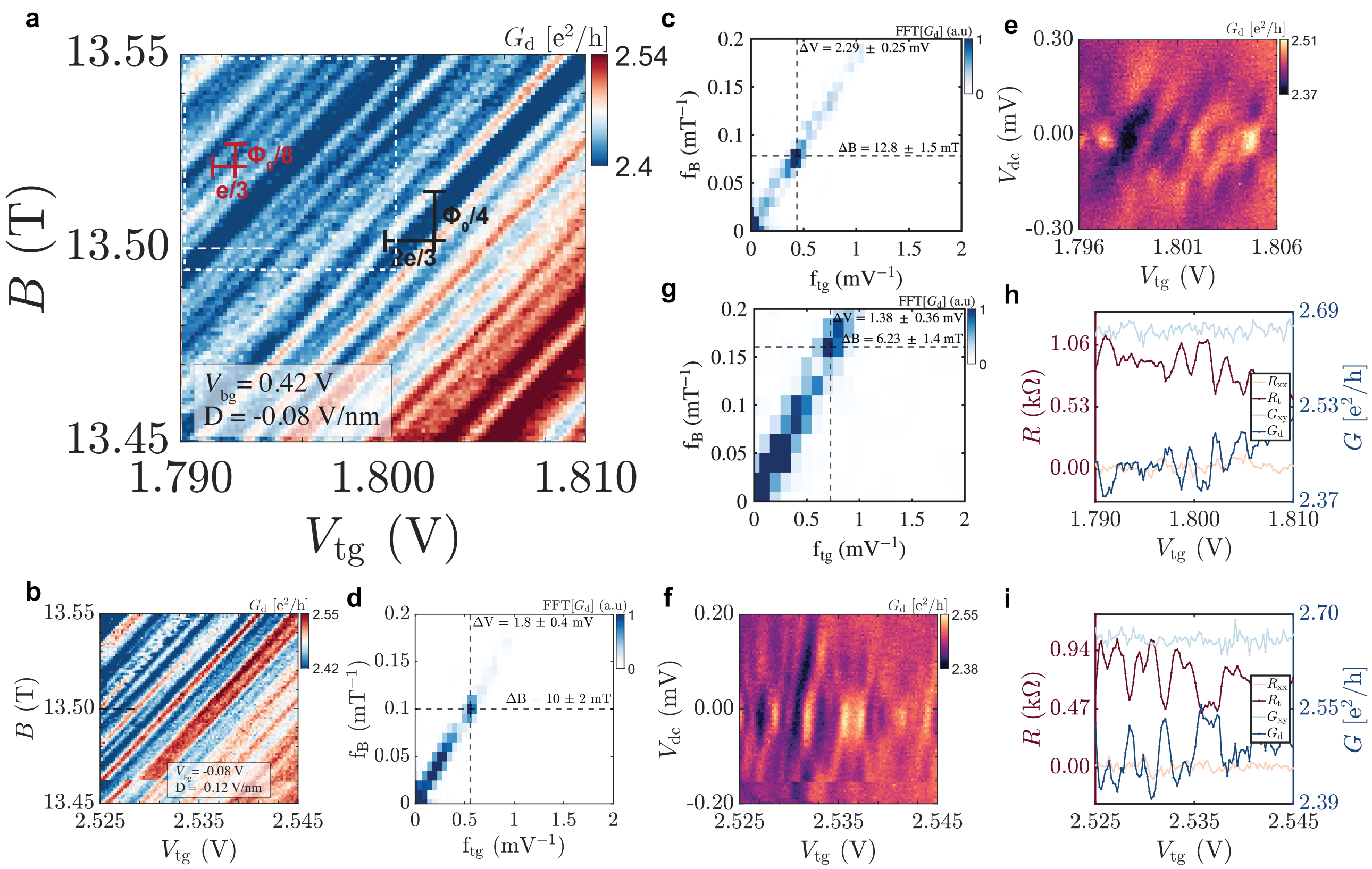

FIG. S18. **Oscillations at $\nu = 8/3$. a-b**, Diagonal conductance G_d oscillations as a function of the TG voltage and magnetic field for filling factor $\nu = 8/3$ at different displacement fields D. The markers in panel **a** highlight the two different oscillation periods present at $\nu = 8/3$. **c-d-g**, 2D-FFT of the oscillations. For panel **g** only the region inside the white dotted line in panel-**a** has been considered.

e-f, Diagonal conductance G_d oscillations as a function of TG voltage and DC bias. **h-i**, Transmitted resistance measured across the AD (R_t in dark red), longitudinal resistance measured outside the AD (R_{xx} in light red), diagonal conductance (G_d in dark blue) and Hall conductance (G_{xy} in light blue) as a function of TG voltage. The oscillations are taken along the dotted lines in their respective panels **a-b**.

H. Renormalization Group Calculation

In this section, we provide a detailed calculation to supplement the discussion of tunnelling quasiparticle charges at $\nu = n + \frac{2}{3}$. First, consider a single $\nu = \frac{2}{3}$ edge which naively consists of a downstream integer boson mode ϕ_1 and an upstream fractional conductance-$\frac{1}{3}$ mode $\phi_{1/3}$. The quadratic action is described by

$$\mathcal{L} = \frac{1}{4\pi}\left[-(\partial_t\phi_I)M_{IJ}\partial_x\phi_J + (\partial_x\phi_I)V_{IJ}(\partial_x\phi_J)\right] \tag{3}$$

where $I, J \in \{1, 1/3\}$, $M = \text{diag}(1, -3)$ and V is a positive-definite 2×2 matrix. The insight of Kane, Fisher, and Polchinski [11] was to consider the charge-neutral operator $\mathcal{O} = e^{i[\phi_1 + 3\phi_{1/3}]}$ which tunnels an electron between the two edge modes. In particular, including this operator in $\mathcal{L}$ with a spatially-random prefactor, they showed that depending on the values of V_{IJ}, this perturbation may be relevant in the renormalization group (RG) sense and lead the system to a "disordered" fixed point with a downstream conductance-$\frac{2}{3}$ mode and an upstream neutral mode.

Now we consider the problem at $\nu = \frac{5}{3}$ which has two downstream integer modes ϕ_1, ϕ_2 (associated with fully filled lowest two LL's) and the upstream $\phi_{1/3}$. The starting point is again a Lagrangian of the form Eq. 3, but now with $I, J \in \{1, 2, 1/3\}$, $M = \text{diag}(1, 1, -3)$ and V a positive-definite 3×3 matrix. There are three important neutral tunneling operators to consider – $\mathcal{O}_{1,2} = e^{i[\phi_{1,2} + 3\phi_{1/3}]}$ which are the analogs of the $\mathcal{O}$ above, and $\tilde{\mathcal{O}} = e^{i[\phi_1 - \phi_2]}$ which tunnnels electrons between the two integer modes.

The long-wavelength physics is determined by the operator with the lowest scaling dimension, which is in turn determined by V_{IJ}. In particular, consider the generalized eigenvectors $u_I^{(j)}$ satisfying $Vu^{(j)} = \lambda_j M u^{(j)}$ where λ_j have the interpretation of eigenmode velocities. It may then be shown that an operator $\mathcal{O}_{\{n\}} = \exp(i\sum n_I\phi_I)$ has scaling dimension

$$\langle\mathcal{O}_{\{n\}}(x)\mathcal{O}_{\{n\}}(0)\rangle \sim x^{-2\Delta_{\{n\}}}, \qquad \Delta_{\{n\}} = \frac{1}{2}\sum_j \frac{\left(\sum_I n_I u_I^{(j)}\right)^2}{\left|(u^{(j)})^T M u\right|} \tag{4}$$

We may use Eq. 4 to find the scaling dimensions of $\mathcal{O}_{1,2}$ and $\tilde{\mathcal{O}}$ discussed above. But doing so requires solving a 3×3 generalized eigenvalue problem which will generally lead to cumbersome expressions.

To build intuition, we first consider cases where $V_{12} = V_{1,1/3} = 0$, so cases where ϕ_1 has no density-density coupling to the other two modes. Following [11], we define $c = \frac{\sqrt{12}V_{2,1/3}}{3V_{22} + V_{1/3,1/3}}$, which must obey $|c| < 1$ following from the positiveness of V. We find that in most of this range, for $c \in [-0.646, 1]$, the most RG-relevant operator is $\tilde{\mathcal{O}}$. But for other values, $c \in [-1, -0.646]$, we have $\mathcal{O}_2$ as the most relevant. It is difficult to make further statements analytically, and without detailed microscopic knowledge of V_{IJ}, we turn to random sampling. We sample $V = X^T X$ with X a 3×3 matrix of i.i.d. real Gaussian entries; we sample V as a Wishart random matrix, which ensures positive-definiteness. The results show that in 75% of the samples, $\tilde{\mathcal{O}}$ is the most RG-relevant operator, while $\mathcal{O}_1$ and $\mathcal{O}_2$ are each the most relevant in 6% of the samples. In a further 5% of the samples, the most relevant operator involves tunnelling more than one electron, and the remaining 8% of the samples have no relevant operators.

We conclude that very likely, $\tilde{\mathcal{O}}$ is the most relevant, so we consider where this leads the system. We start by focusing on ϕ_1 and ϕ_2 only, where a well-established procedure exists [12]. We write the 2×2 coupling of $\phi_{1,2}$ in V as $V = v\mathbb{I}_2 + \delta V$ with δV traceless. Then, we fermionize these two edge modes to yield

$$\mathcal{L} = i\psi_J^\dagger(\partial_t - v\partial_x)\psi_J + \xi(x)\psi_2^\dagger\psi_1 + \xi^*(x)\psi_1^\dagger\psi_2 + \delta V_{IJ}(\psi_I^\dagger\psi_I)(\psi_J^\dagger\psi_J) \tag{5}$$

The advantage is that now the tunneling operator $\tilde{O}$ takes on the simple form $\psi_2^\dagger\psi_1$, which we included above with a spatially random pre-factor $\xi(x)$. Finally, we write $\psi_a(x) = U_{ab}(x)\tilde{\psi}_b(x)$ with $U(x)$ a unitary chosen such that

$$\partial_x U(x) = \frac{i}{v}\begin{pmatrix} 0 & \xi^*(x) \\ \xi(x) & 0 \end{pmatrix} U(x) \tag{6}$$

Re-writing Eq. 5 using this gives

$$\mathcal{L} = i\tilde{\psi}_J^\dagger(\partial_t - v\partial_x)\tilde{\psi}_J + \mathcal{O}((\tilde{\psi}^\dagger\tilde{\psi})^2) \tag{7}$$

In this way, we have gauged away the tunnelling. The typical magnitude of $\xi(x)$ sets a lengthscale l_{dis} beyond which the unitaries $U(x)$ are uncorrelated. Rewriting the density operators in the new language gives

$$\begin{aligned}\rho_1 = \frac{1}{2\pi}\partial_x\phi_1 = \psi_1^\dagger\psi_1 = U_{1a}(x)U_{1b}^*(x)\tilde{\psi}_b\tilde{\psi}_a = \frac{1}{4\pi}(\partial_x\tilde{\phi}_1 + \partial_x\tilde{\phi}_2)+ \\ +R_1(x)\partial_x\tilde{\phi}_1 + R_2(x)\partial_x\tilde{\phi}_2 + R_3(x)e^{i[\tilde{\phi}_1 - \tilde{\phi}_2]} + \text{h.c.}\end{aligned} \tag{8}$$

Where $\tilde{\phi}_{1,2}$ are bosonizations of $\tilde{\psi}_{1,2}$ and all $R_k(x)$ are taken as spatially random with zero mean, correlated only on scales up to $l_{\rm dis}$. The density ρ_2 obeys an analogous expression. After treating randomness, the expressions for physical densities in terms of the new bosons include purely random factors up to the constraint that the total charge density in $\tilde{\phi}_{1,2}$ is equal to that in $\phi_{1,2}$. Re-introducing the fractional mode, the edge is now described by (dropping the tilde on $\phi_{1,2}$)

$$\begin{aligned}
\mathcal{L} &= \mathcal{L}_0 + \mathcal{L}_{\rm random} \\
\mathcal{L}_0 &= \frac{1}{4\pi}\left[-(\partial_t\phi_I)M_{IJ}\partial_x\phi_J + (\partial_x\phi_I)V_{IJ}(\partial_x\phi_J)\right] \\
V_{IJ} &= \begin{pmatrix} v & 0 & t \\ 0 & v & t \\ t & t & u \end{pmatrix} \\
\mathcal{L}_{\rm random} &= R_1(x)\partial_x\phi_1\partial_x\phi_3 + R_2(x)\partial_x\phi_2\partial_x\phi_3 + R_3(x)\partial_x\phi_3\left(e^{i[\phi_1-\phi_2]} + \text{h.c.}\right) \\
&\quad + R_4(x)\partial_x\phi_1\partial_x\phi_2 + R_5(x)e^{i[\phi_1+3\phi_3]} + \dots
\end{aligned} \tag{9}$$

Here $M = \text{diag}(1,1,-3)$ and crucially Eq. 8 enforces this particular form of V_{IJ}. The upshot is that now all operators in $\mathcal{L}_{\rm random}$ can be shown to have scaling dimension $\Delta > \frac{3}{2}$, i.e., they are RG-irrelevant, regardless of the values in V_{IJ}. For example, we may use Eq. 4 to find that the dimension of the operator multiplied by $R_5(x)$ is $\Delta_5 = \frac{1}{4} + \frac{1}{4}\left(7(3u+v) - 24t\right)/\sqrt{(3u+v)^2 - 24t^2}$. The positive-definitiveness of V_{IJ} implies that $uv \geq 2t^2$ which may be used to show $\Delta_5 \geq \frac{3}{2}$ always. One may also show that $\Delta_1 = \Delta_2 = \Delta_3 = \Delta_4 = 2$. This means that there can be no relevant operator coupling the fractional mode to the integer edge. The strong hybridization between integer modes blocks the Kane-Fisher-Polchinski mechanism and leaves an upstream conductance-$\frac{1}{3}$ mode intact.

By analogous reasoning, one may consider $\nu = n + \frac{2}{3}$ for $n > 0$ an integer. Then we have $n+1$ integer modes $\phi_1, \dots, \phi_{n+1}$ and if we assume they can all couple strongly, the integer modes will flow into a disordered fixed point while the fractional mode would remain decoupled. But as noted above, some of these couplings may be very weak in practice, and thus for some n, this physics could only be visible at temperatures well below what is used in experiments. Alternatively, if the tunneling is weak, the length $l_{\rm dis}$ over which $R_k(x)$ are uncorrelated becomes large, and the terms in $\mathcal{L}_{\rm random}$ no longer have random pre-factors which means they can become relevant if they have $\Delta \leq 2$, which is a weaker requirement than the $\Delta \leq \frac{3}{2}$ needed for an operator with a random pre-factor to be relevant.

This later point is crucial for understanding the physics at $\nu = 2 + \frac{2}{3}$, where the coupling between the two modes at the fractional edge and the two integer modes is expected to be weak, and any possibly RG-relevant operator coupling the fractional and integer edges will only become important at low temperatures. We may think of the $\nu = 2 + \frac{2}{3}$ case in the framework of Eq. 3 with $M = \text{diag}(1,1,1,-3)$ where $I = 1,2$ refer to the integer modes and $I = 3,4$ is the fractional edge. The physical separation of the integer and fractional edges would be encoded by demanding that $V_{(1\text{ or }2)(3\text{ or }4)}$ is small, which makes it likely that the most RG-relevant operator involving the fractional edge is simply the one discussed by Kane, Fisher and Polchinski, leading to the expectation that the physics at $\nu = \frac{8}{3}$ will be similar to $\nu = \frac{2}{3}$, where we expect the presence of a downstream charged mode and an upstream neutral mode.

If the Kane-Fisher-Polchinski mechanism is blocked (as is the case at $\nu = \frac{5}{3}$), it is evident that $q = \frac{1}{3}e$ is the tunneling charge with the lowest scaling dimension. But the lowest tunneling charge is not immediately obvious in the case where the system flows to the Kane-Fisher-Polchniski "disordered" fixed point, where both operators with $q = \frac{1}{3}e$ and with $q = \frac{2}{3}e$ have equal scaling dimension, and one must compare tunneling matrix elements instead. Without a detailed microscopic model of the edge, making any definitive statements about those is difficult. One line of reasoning might argue that the tunneling of the lower charge should have a larger amplitude. Alternatively, at the disordered fixed point of [11], it is natural to think about the "total charge" mode $\phi_\rho = \sqrt{\frac{3}{2}}(\phi_1 + \phi_{1/3})$ and the "neutral" mode $\phi_\sigma = \sqrt{\frac{1}{2}}(\phi_1 + 3\phi_{1/3})$. To tunnel a charge-$\frac{2}{3}$ anyon, we may use the operator $e^{i(\phi_1+\phi_{1/3})} = \exp(i\sqrt{2/3}\phi_\rho)$, while the tunneling of a charge-$\frac{1}{3}$ requires $e^{i\phi_{1/3}} = \exp(i\phi_\sigma/\sqrt{2})\exp(-i\phi_\rho/\sqrt{6})$. Thus only the tunneling of the smaller $q = \frac{1}{3}$ needs to use the neutral mode ϕ_σ – a small tunneling amplitude for the field ϕ_σ (which at the fixed point is independent of ϕ_ρ) could be the cause of suppression of the tunneling amplitude for the smaller charge, although details of why such a suppression might appear remain unclear.

A more detailed microscopic of the above would of course be ideal – but the above effective-theory analysis still importantly (1) identifies an important difference in the edge theories between $\nu = \frac{5}{3}$ and $\nu = \frac{2}{3}, \frac{8}{3}$, (2) explains the tunneling charge at $\nu = \frac{5}{3}$ and (3) reduces the question of why $q = \frac{2}{3}e$ is seen at $\nu = \frac{8}{3}$ down to questions about $\nu = \frac{2}{3}$ which has also been explored in GaAs as discussed above.

To conclude this section, we analyse the relevant length-scales in the experiment. First, the antidot circumference is $\pi D_{AD} \sim 1$ μm and the magnetic length is $l_B \sim 6.5$ nm at $B \approx 13$ T. The magnetic length l_B serves as a UV cutoff of the theory, and the fact that the antidot is large compared to that justifies the application of RG-flow based ideas.

The next crucial lengthscale to consider is the thermal length $L_T \sim \frac{\hbar v}{k_B T}$ with $v \approx 10^5$ ms^{-1} a typical edge mode velocity. We estimate $L_T \approx 50$ μm, which is large compared to the antidot's size. At $\nu = \frac{2}{3}$ and $\nu = \frac{8}{3}$, RG-relevant couplings drive the system to a fixed point with the fractional part of the conductance quantized to $\frac{2}{3}\frac{e^2}{h}$. But the situation is different at $\nu = \frac{5}{3}$, where we argue that the fixed point has two downstream integer modes and an upstream fractional mode. If the modes were to not reach equiblirium, we would see a conductance $\sigma_{xy} = (2 + \frac{1}{3})\frac{e^2}{h} \neq \frac{5}{3}\frac{e^2}{h}$. But while no terms in $\mathcal{L}_{\text{random}}$ in Eq. 9 are RG-relevant – this does not mean that these terms cannot lead the system to equilibrium, it simply means that the relevant equilibration length will diverge at low temperatures. In particular, a scaling analysis suggests that $L_{\text{eq}} \sim l_B\,(L_T/l_B)^{2\Delta-2}$ where Δ is the dimension of the most RG-relevant operator coupling the integer and fractional modes [12–14]. As pointed out in [14], this only holds when $\Delta \geq \frac{3}{2}$ and the scaling is more subtle for RG-relevant operators. But all operators coupling to the fractional mode in Eq. 9 have $\Delta \geq \frac{3}{2}$, but in particular (depending on V_{IJ}), Δ_5 may be very close to this bound. If we write $\Delta = \frac{3}{2} + \epsilon$, we find $L_{\text{eq}} \sim K L_T (L_T/l_B)^{2\epsilon}$. The dimensionless pre-factor K is fixed by microscopic details of the interactions between the modes, the calculation of which we do not attempt.

In cases where ϵ is not too large (where the scaling dimension Δ_5 is relatively small), this equilibration length is comparable to the thermal length – while far too large to display a good degree of equilibration on the antidot itself, it might be able to bring the edge modes to equilibrium when traveling around the entire device, which is the important part needed to see an approximately quantized diagonal Hall conductance in experiment.

---

[1] J. Kim, H. Dev, R. Kumar, A. Ilin, A. Haug, V. Bhardwaj, C. Hong, K. Watanabe, T. Taniguchi, A. Stern, and Y. Ronen, Aharonov–Bohm interference and statistical phase-jump evolution in fractional quantum Hall states in bilayer graphene, Nat. Nanotechnol. **19**, 1619 (2024).

[2] B. M. Hunt, J. I. A. Li, A. A. Zibrov, L. Wang, T. Taniguchi, K. Watanabe, J. Hone, C. R. Dean, M. Zaletel, R. C. Ashoori, and A. F. Young, Direct measurement of discrete valley and orbital quantum numbers in bilayer graphene, Nat. Commun. **8**, 1 (2017).

[3] J. Li, Y. Tupikov, K. Watanabe, T. Taniguchi, and J. Zhu, Effective Landau Level Diagram of Bilayer Graphene, Phys. Rev. Lett. **120**, 047701 (2018).

[4] R. Kumar, A. Haug, J. Kim, M. Yutushui, K. Khudiakov, V. Bhardwaj, A. Ilin, K. Watanabe, T. Taniguchi, D. F. Mross, and Y. Ronen, Quarter- and half-filled quantum Hall states and their topological orders revealed by daughter states in bilayer graphene, Nat. Commun. **16**, 1 (2025).

[5] J. Martin, B. E. Feldman, R. T. Weitz, M. T. Allen, and A. Yacoby, Local Compressibility Measurements of Correlated States in Suspended Bilayer Graphene, Phys. Rev. Lett. **105**, 256806 (2010).

[6] Y. Ronen, T. Werkmeister, D. Haie Najafabadi, A. T. Pierce, L. E. Anderson, Y. J. Shin, S. Y. Lee, Y. H. Lee, B. Johnson, K. Watanabe, T. Taniguchi, A. Yacoby, and P. Kim, Aharonov–Bohm effect in graphene-based Fabry–Pérot quantum Hall interferometers, Nat. Nanotechnol. **16**, 563 (2021).

[7] C. Déprez, L. Veyrat, H. Vignaud, G. Nayak, K. Watanabe, T. Taniguchi, F. Gay, H. Sellier, and B. Sacépé, A tunable Fabry–Pérot quantum Hall interferometer in graphene, Nat. Nanotechnol. **16**, 555 (2021).

[8] T. Werkmeister, J. R. Ehrets, M. E. Wesson, D. H. Najafabadi, K. Watanabe, T. Taniguchi, B. I. Halperin, A. Yacoby, and P. Kim, Anyon braiding and telegraph noise in a graphene interferometer, Science **388**, 730 (2025).

[9] N. L. Samuelson, L. A. Cohen, W. Wang, S. Blanch, T. Taniguchi, K. Watanabe, M. P. Zaletel, and A. F. Young, Anyonic statistics and slow quasiparticle dynamics in a graphene fractional quantum Hall interferometer, arXiv 10.48550/arXiv.2403.19628 (2024), 2403.19628.

[10] N. Moreau, S. Faniel, F. Martins, L. Desplanque, X. Wallart, S. Melinte, V. Bayot, and B. Hackens, Revisiting Coulomb diamond signatures in quantum Hall interferometers, Phys. Rev. B **105**, 115144 (2022).

[11] C. L. Kane, M. P. A. Fisher, and J. Polchinski, Randomness at the edge: Theory of quantum hall transport at filling ν=2/3, Phys. Rev. Lett. **72**, 4129 (1994).

[12] C. L. Kane and M. P. A. Fisher, Impurity scattering and transport of fractional quantum hall edge states, Phys. Rev. B **51**, 13449 (1995).

[13] K. K. W. Ma and D. E. Feldman, Thermal equilibration on the edges of topological liquids, Physical Review Letters **125**, 10.1103/physrevlett.125.016801 (2020).

[14] I. V. Protopopov, Y. Gefen, and A. D. Mirlin, Transport in a disordered ν=xn–23-6bv fractional quantum Hall junction, Ann. Phys. **385**, 287 (2017).
