# Supplementary material for: Quantum Hall antidot as a fractional coulombmeter
Source: Nat Phys. 2026 Aug 14;22(10):1587–93. doi: 10.1038/s41567-026-03412-2 (PMC13658039; doi:10.1038/s41567-026-03412-2)
Supplement: Supplementary file 2 [file 41567_2026_3412_MOESM2_ESM.pdf]

nature portfolio

# Peer Review File

# Quantum Hall Antidot as a Fractional Coulombmeter

Corresponding Author: Professor Mitali Banerjee

This manuscript has been previously reviewed at another journal. This document only contains information relating to versions considered at Nature Physics.

Version 0:

Reviewer comments:

Reviewer #1

(Remarks to the Author)
I have read the revised manuscript together with the authors' response to the first-round reports. The fabrication quality and level of control achieved in this device remain highly impressive and constitute a significant technical accomplishment. In that respect, the work appeared aligned with the standards of Nature Nanotechnology, assuming the authors replied to my initial concerns.

However, for Nature Physics, it is less clear what the central new physics result is and whether the advance is sufficiently compelling for that venue. In addition, the response to the main concern regarding the determination of the oscillation period is not fully satisfactory. This point is central, since the extraction of the effective fractional charge relies directly on it and therefore underpins the principal claim of the manuscript.

The authors explain that, for most of the fractional filling factors, the oscillation period was determined directly from the conductance maps because the Fourier transforms do not show well-resolved peaks. This approach is acceptable in principle, provided that it is described in a clear, systematic, and reproducible manner. At present, however, several conductance maps, for example Fig. 3b-f and Fig. S9g-I, show clearly that the period is not constant and can vary substantially depending on which conductance maxima are selected. This likely explains why the Fourier transforms do not yield sharp peaks, but rather broad features spread over a range of frequencies. As a consequence, the precision of the extracted charge values appears uncertain.

A related concern is that the manuscript does not specify which oscillations were selected for the period extraction. Multiple maxima could plausibly be used in the conductance maps, yet the analysis procedure is not explicitly indicated. At a minimum, the relevant maxima should be marked directly on the figures so that the extraction method can be followed. In its current form, the analysis cannot be independently reproduced from the data shown.

There is also an issue with the discussion of resolution limits. The authors attribute the absence of a single 2D-FFT peak mainly to two factors: (1) the narrow width of the plateau, which limits the number of oscillations, and (2) the small oscillation period, which is said to be affected by the discrete magnetic-field and gate-voltage step sizes. This explanation is not convincing as presently formulated. The conductance maps do not appear to suffer from insufficient experimental resolution. Standard magnetic-field resolution is typically of the order of 1 mT or better, whereas the relevant oscillation periods are on the order of several tens of mT, so magnet step size should not be a limiting factor. Moreover, the statement that the "minimal frequency resolution is set by the step sizes in the magnetic field and gate voltage" is not correct. Rather, as indicated by the authors' own equation in Section SI-E, the minimal frequency resolution is determined by the total size of the magnetic-field and gate-voltage windows.

Overall, in view of the limited accuracy in the determination of the oscillation periods, the substantial prior literature on quantum Hall antidots in both GaAs and graphene, the extensive existing body of work on fractional charge measurements via shot noise, and the difficulty in identifying a sufficiently clear and distinct new physics message, this work does not appear suitable for publication in Nature Physics.

Reviewer #3

(Remarks to the Author)
I do not have much to add to my previous review. The authors' response to my comments is satisfactory, and so I recommend publication. I think this paper would be suitable for Nature Nanotechnology, but it also fits Nature Physics, and Nature Physics might be a more logical choice of a journal. The only new point I would like to make concerns a criticism by Referee 2. I do not think that a comparison with what was achieved in GaAs is a valid approach here, though I do think that the progress beyond what was achieved in GaAs is significant. More importantly, graphene, especially, bilayer graphene is different from GaAs both in terms of physics and in terms of what experimental tehcniques are feasible and how they work.

Version 1:

Reviewer comments:

Reviewer #1

(Remarks to the Author)
The authors have improved and clarified their frequency analysis by performing a careful statistical analysis of the conductance oscillations. The resulting histograms, for example in Fig. S6, show well-defined peaks consistent with the Fourier transforms. The reader can now independently assess the accuracy of the period determination. My concerns about the methodology are therefore resolved.

**Open Access** This Peer Review File is licensed under a Creative Commons Attribution 4.0 International License, which permits use, sharing, adaptation, distribution and reproduction in any medium or format, as long as you give appropriate credit to the original author(s) and the source, provide a link to the Creative Commons license, and indicate if changes were made.
In cases where reviewers are anonymous, credit should be given to 'Anonymous Referee' and the source.
The images or other third party material in this Peer Review File are included in the article’s Creative Commons license, unless indicated otherwise in a credit line to the material. If material is not included in the article’s Creative Commons license and your intended use is not permitted by statutory regulation or exceeds the permitted use, you will need to obtain permission directly from the copyright holder.
To view a copy of this license, visit https://creativecommons.org/licenses/by/4.0/

**Reviewer #1 (Remarks to the Author):**

I have read the revised manuscript together with the authors' response to the first-round reports. The fabrication quality and level of control achieved in this device remain highly impressive and constitute a significant technical accomplishment. In that respect, the work appeared aligned with the standards of Nature Nanotechnology, assuming the authors replied to my initial concerns.

We thank the reviewer for acknowledging the complexity of this experiment and for recognizing the level of fabrication quality and technical control achieved in our device. We are glad that these aspects are appreciated, and we have done our best to address all of the reviewer's concerns, as detailed in the responses below.

However, for Nature Physics, it is less clear what the central new physics result is and whether the advance is sufficiently compelling for that venue. In addition, the response to the main concern regarding the determination of the oscillation period is not fully satisfactory. This point is central, since the extraction of the effective fractional charge relies directly on it and therefore underpins the principal claim of the manuscript.

The authors explain that, for most of the fractional filling factors, the oscillation period was determined directly from the conductance maps because the Fourier transforms do not show well-resolved peaks. This approach is acceptable in principle, provided that it is described in a clear, systematic, and reproducible manner. At present, however, several conductance maps, for example Fig. 3b-f and Fig. S9g-l, show clearly that the period is not constant and can vary substantially depending on which conductance maxima are selected. This likely explains why the Fourier transforms do not yield sharp peaks, but rather broad features spread over a range of frequencies. As a consequence, the precision of the extracted charge values appears uncertain.

A related concern is that the manuscript does not specify which oscillations were selected for the period extraction. Multiple maxima could plausibly be used in the conductance maps, yet the analysis procedure is not explicitly indicated. At a minimum, the relevant maxima should be marked directly on the figures so that the extraction method can be followed. In its current form, the analysis cannot be independently reproduced from the data shown.

We thank the reviewer for their constructive comment, which touches on a central aspect of our analysis. We address below the concern regarding the extraction of the oscillation period and the reproducibility of our procedure.

We acknowledge that, due to the small extent of the FQHE plateaus in both magnetic field and gate voltage, the oscillation period can slightly vary across the conductance map. This non-uniformity is one of the reasons why the 2D-FFT does not always yield sharp, well-resolved peaks, but instead produces broader spectral features. An additional contributing factor is that the top gate voltage simultaneously controls the oscillations (the bulk density), and the coupling to the extended edge states, which can modify the oscillation period. In particular, if the symmetry of the oscillations in one of

the two tunneling regions is more strongly affected by this coupling, this could lead to a further variation across the map.

To extract the period in a systematic and consistent manner, we select line cuts at the center of the conductance map, both along the magnetic-field axis (horizontal) and along the gate-voltage axis (vertical), and average the extracted periods over these central lines. This choice minimizes the influence of edge effects and regions where the coupling asymmetry is most pronounced, and we believe provides a representative value for the oscillation period within the plateau.

We recognize that this procedure was not sufficiently transparent in the previous version and we thank the referee to point that out.

Following the reviewer's suggestion, we have improved our procedure using a more systematic and less arbitrary way for extracting the period and its associated uncertainty. The improved method is consistent with our previous findings. The procedure is described in detail in the Supplementary Material Section E and summarised below.

1. We first identify the relevant oscillation period visually from the 2D pyjama plot. For each dataset, the oscillations used for period identification are explicitly marked. We preferentially select oscillations near the centre of the gate voltage and magnetic field range, as these are least affected by changes in coupling strength or filling factor arising from deviations from the plateau.
2. For each value of top-gate voltage and magnetic field, we identify peaks in the oscillations using the MATLAB *findpeaks* function. The period is then estimated from the pairwise differences between all detected peaks, and the resulting values are compiled into a histogram. The bin width is set to twice the measurement step size, reflecting the systematic uncertainty of the peak-finding procedure.
3. The histogram is fitted with a Gaussian function to extract the mean value.
4. Using the mean value obtained in step 3, we identify the corresponding peak in the 2D-FFT closest to that value. To suppress low-frequency contributions, as is standard practice in interference experiments, a linear plane is subtracted from the data prior to the FFT.
5. A line cut through the 2D-FFT at the identified peak frequency is fitted with a Gaussian. The mean and variance of this fit are used as the final estimates of the period and its uncertainty, respectively.

The error obtained in this way accounts for the statistical error of the oscillations. In two cases, $\nu = 7/3$ and the small oscillations at $\nu = 8/3$ we believe this error underestimates the real uncertainty as it does not take into account the difficulty in finding the correct peak. So for this two cases we have taken as the error on the period twice the statistical uncertainty.

Using this new method we find a smaller error for $\nu = 4/3$, 5/3 and the large oscillation at 8/3; a comparable error at 3/5 and the small 8/3 oscillation and a larger error for 2/3 and 7/3. We have updated Fig. 4 and Fig. 5 with the values found using this procedure.

Additional comments on this procedure:

- For $\nu = 7/3$ at B = 8 T, the histogram overestimates the period, which we attribute to the low visibility of the oscillations and the consequent difficulty in reliably identifying peaks with *findpeaks*. In this case, we rely solely on the periods indicated by the lines drawn on the plot and apply the procedure from step 4 onward. We note that the uncertainty obtained in this way underestimates the true error, as it reflects only the width of the FFT peak and does not account for the uncertainty in locating the oscillations. We therefore adopt a conservative estimate of twice this value to reflect the higher uncertainty in this measurement.

- For $\nu = 8/3$, the analysis was performed separately in two distinct gate-voltage regions, as applying the procedure to the full range causes the histogram to return an average of the two distinct periodicities present.

- For $\nu = 4/3$ at B = 8 T (Figure S7b) we observe in the histogram a second peak. We attribute it to an occasional misdetection by the *findpeaks* function. When the algorithm fails to identify a true peak, the apparent period doubles. In such cases, we use the period extracted from the conductance map to recover the correct frequency.

- For 2/3 and 3/5 the magnetic field histogram is broad. However, we non theless observe the the variance of the hisotgram is comparable to the final error of the 2D-FFT.

We believe these additions significantly improve the clarity and reproducibility of our period extraction procedure, and therefore of the effective fractional charge values reported in the manuscript.

There is also an issue with the discussion of resolution limits. The authors attribute the absence of a single 2D-FFT peak mainly to two factors: (1) the narrow width of the plateau, which limits the number of oscillations, and (2) the small oscillation period, which is said to be affected by the discrete magnetic-field and gate-voltage step sizes. This explanation is not convincing as presently formulated. The conductance maps do not appear to suffer from insufficient experimental resolution. Standard magnetic-field resolution is typically of the order of 1 mT or better, whereas the relevant oscillation periods are on the order of several tens of mT, so magnet step size should not be a limiting factor. Moreover, the statement that the "minimal frequency resolution is set by the step sizes in the magnetic field and gate voltage" is not correct. Rather, as indicated by the authors' own equation in Section SI-E, the minimal frequency resolution is determined by the total size of the magnetic-field and gate-voltage windows.

We thank the reviewer for pointing this out and for the careful reading of our manuscript. We agree that our original statement was incorrectly formulated: as written in the SI-E the minimal frequency resolution is indeed determined by the total size of the magnetic-field and gate-voltage windows ($N_B \cdot dB$ or $N_V \cdot dV$), and not by the step sizes dB and dV themselves.

Regarding the experimental resolution: our minimal step in magnetic field is dB = 1 mT and in gate voltage is dV = 0.1 mV. For the smallest fractional periods, such as the 1/3 states in gate voltage and large-N states in magnetic field (e.g., 8/3), this allows us to resolve approximately 10 data points per oscillation period, which is sufficient for reliable FFT analysis.

However, in cases where we cannot rely solely on the FFT, the discrete step size becomes a relevant limitation, not for the frequency resolution per se, as the reviewer suggests, but for our ability to discriminate between closely spaced periodicities. This is the reason we stated the measurement resolution as a limiting factor, though we acknowledge it was cited in the wrong context.

That said, as discussed in the final paragraph of SI-E, the dominant source of uncertainty in our analysis remains the finite size of the FQHE plateaus. This limits the number of observable oscillations and, consequently, the frequency resolution of the 2D-FFT

Overall, in view of the limited accuracy in the determination of the oscillation periods, the substantial prior literature on quantum Hall antidots in both GaAs and graphene, the extensive existing body of work on fractional charge measurements via shot noise, and the difficulty in identifying a sufficiently clear and distinct new physics message, this work does not appear suitable for publication in Nature Physics.

We thank the reviewer for their detailed assessment, but we respectfully disagree with their conclusion regarding the novelty and significance of our work.

Regarding the prior literature on quantum Hall antidots, we note that the existing body of work is in fact very limited. Only few studies have reported antidot experiments in the fractional quantum Hall effect regime, namely those by Goldman at al. (*Science* 267, 1010 (1995)) and Kou et al. (*Phys. Rev. Lett.* 108, 256803 (2012)), and these were carried out in GaAs. In graphene specifically, the literature on antidots is extremely sparse, with only one group having reported antidot measurements in the integer quantum Hall effect regime using suspended or etched graphene, and with only few oscillations observed in the fractional regime, at $\nu = 2/3$ by S. M. Mills et al. (*Phys. Rev. Lett.* 125, 227701 (2020)). Our work therefore represents a thorough and systematic study of antidot physics in the FQHE in graphene, going beyond these prior observations.

Additionally as also written by R3, bilayer graphene and GaAs are fundamentally different platforms, and one should not a priori expect the same physical behavior in the two systems.

For these reasons, we maintain that our work offers a genuinely new contribution to the field, both in terms of the experimental platform and the physical insight it provides.

**Reviewer #3 (Remarks to the Author):**

I do not have much to add to my previous review. The authors' response to my comments is satisfactory, and so I recommend publication. I think this paper would be suitable for Nature Nanotechnology, but it also fits Nature Physics, and Nature Physics might be a more logical choice of a journal. The only new point I would like to make concerns a criticism by Referee 2. I do not think that a comparison with what was achieved in GaAs is a valid approach here, though I do think that the progress beyond what was achieved in GaAs is significant. More importantly, graphene, especially, bilayer graphene is different from GaAs both in terms of physics and in terms of what experimental tehcniques are feasible and how they work.

We thank the reviewer for their positive assessment of our work and for recommending publication. We are pleased that our responses to the first-round comments were found satisfactory.

We also thank the reviewer for their remark regarding the comparison of BG with GaAs raised by R2. We fully agree with the reviewer's perspective on this point. While we believe that the progress achieved in our work goes significantly beyond what has been demonstrated in GaAs systems, we concur that a direct comparison is not straightforward and may not be the most appropriate framework for evaluating our results.
